# Lymphatic Endothelial Cell Junctions: Molecular Regulation in Physiology and Diseases

**DOI:** 10.3389/fphys.2020.00509

**Published:** 2020-05-29

**Authors:** Feng Zhang, Georgia Zarkada, Sanjun Yi, Anne Eichmann

**Affiliations:** ^1^State Key Laboratory of Ophthalmology, Zhongshan Ophthalmic Center, Sun Yat-sen University, Guangzhou, China; ^2^Department of Cellular and Molecular Physiology, Cardiovascular Research Center, Yale School of Medicine, Yale University, New Haven, CT, United States; ^3^INSERM U970, Paris Cardiovascular Research Center, Paris, France

**Keywords:** lymphatic vessel, endothelial junction, button-like junction, zipper-like junction, VE-cadherin, VEGFR2 signaling

## Abstract

Lymphatic endothelial cells (LECs) lining lymphatic vessels develop specialized cell-cell junctions that are crucial for the maintenance of vessel integrity and proper lymphatic vascular functions. Successful lymphatic drainage requires a division of labor between lymphatic capillaries that take up lymph via open “button-like” junctions, and collectors that transport lymph to veins, which have tight “zipper-like” junctions that prevent lymph leakage. In recent years, progress has been made in the understanding of these specialized junctions, as a result of the application of state-of-the-art imaging tools and novel transgenic animal models. In this review, we discuss lymphatic development and mechanisms governing junction remodeling between button and zipper-like states in LECs. Understanding lymphatic junction remodeling is important in order to unravel lymphatic drainage regulation in obesity and inflammatory diseases and may pave the way towards future novel therapeutic interventions.

## Introduction

The lymphatic system plays pivotal roles in fluid balance, immune cell trafficking and lipid uptake (Petrova and Koh, 2018; Cifarelli and Eichmann, 2019; Jackson, 2019b), and lack or malfunction of lymphatic vessels leads to edema, disturbed immune responses and lipid malabsorption (Alitalo, 2011; Venero Galanternik et al., 2016; Escobedo and Oliver, 2017). The lymphatic vasculature is present in most organs, and consists of a complex branched network of capillaries (also called initial lymphatics), collecting vessels and lymph nodes. The blind-ended initial lymphatics are lined by a single layer of oak leaf-shaped endothelial cells, which are characterized by a sparse and highly perforated basement membrane, numerous anchoring filaments and lack of perivascular cell coverage (Gerli et al., 2000; Pflicke and Sixt, 2009; Tammela and Alitalo, 2010; Schulte-Merker et al., 2011). Fluids, macromolecules and immune cells that extravasate from blood capillaries are drained from the interstitium by the initial lymphatics. The absorbed fluid, known as lymph, flows towards collecting lymphatics, which are characterized by an intact basement membrane and are covered with smooth muscle cells. The collecting lymphatic vessels contain intraluminal valves to prevent the backflow of lymph, which is transported through progressively larger collecting vessels and lymph nodes, and eventually returns back to the blood circulation via lymphatic connections to major veins (Tammela and Alitalo, 2010; Schulte-Merker et al., 2011; Petrova and Koh, 2018). Of note, the lymphatic system has been known since the mid-1660s to transport dietary lipids from the intestine to the blood circulation (Suy et al., 2016). Dietary fats are absorbed by intestinal enterocytes and assembled into triglyceride-enriched particles called chylomicrons. Thereafter, chylomicrons are taken up by initial intestinal lymphatics called lacteals and transported into the blood circulation via the lymphatic system (Randolph and Miller, 2014; Bernier-Latmani and Petrova, 2017; Petrova and Koh, 2018; Cifarelli and Eichmann, 2019).

**FIGURE 1:**
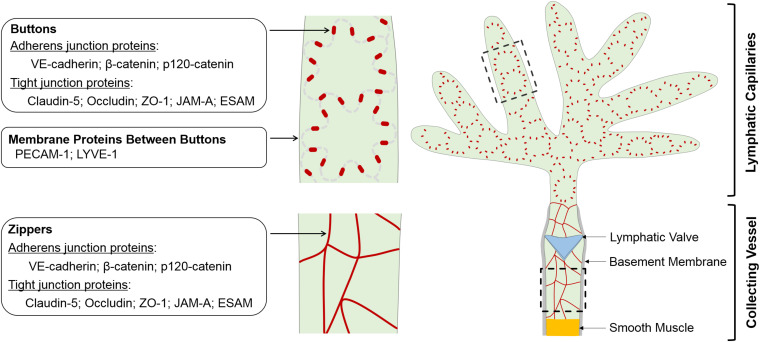
Schematic of morphology, localization and protein components of LEC junctions. LECs lining initial and collecting lymphatics develop different cell-cell junctions to execute uptake and transport of lymph, respectively. The specialized discontinuous buttons in initial lymphatics serve as anchoring sites at the sides of interdigitated flaps of adjacent oak leaf-shaped LECs, and the loosely apposed border regions between buttons are entry sites for fluids, chylomicrons and immune cells. The endothelium of collecting vessels has continuous zippers that prevent vascular leak and allow transport of lymph, a process that is aided by smooth muscle cell coating and intraluminal valves. Adherens junction- and tight junction-associated proteins are enriched in both buttons and zippers. In mature initial lymphatics, most PECAM-1 and LYVE-1 are distributed at the tips of flaps between buttons. LEC, lymphatic endothelial cell.

Blood and lymphatic vessels are both lined with endothelial cells, which are connected by cell-cell junctions. Blood endothelial cell (BEC) junctions include adherens junctions expressing VE-cadherin, tight junctions expressing claudins and gap junctions characterized by connexin expression. BEC junctions are involved in a range of blood vascular processes, such as vasculogenesis, angiogenesis, vessel leak and leukocyte extravasation (Dejana et al., 2009b; Muller, 2015; Okamoto and Suzuki, 2017; Szymborska and Gerhardt, 2018). LEC junctions, on the other hand, have unique structural characteristics reflecting the specific demands of the lymphatic vasculature. In lymphatic capillaries, discontinuous button-like junctions (buttons) (Figure 1) endow these vessels with high permeability to allow the entry of interstitial fluid, macromolecules, lipids and immune cells, while collecting lymphatics have continuous zipper-like junctions (zippers) that strengthen the vascular barrier to allow lymph transport. Buttons and zippers are named based on their distinct morphology to distinguish focal sites of intercellular adhesions (buttons) in lymphatic capillaries, and mature junctions (zippers) in lymphatic collectors that are also present between endothelial cells of other blood vessels (Baluk et al., 2007).

An increasing number of studies have now shown that lymphatic drainage requires properly organized LEC junctions that are dynamically remodeled; yet, the molecular basis of LEC junction remodeling in development and disease is at an early stage of investigation. Here, we discuss the current state-of-the-art on the molecular organization of LEC junctions in physiological and pathological contexts.

## General Histology of LEC Junctions

The oak leaf-shaped LECs of initial lymphatics have overlapping flaps that have long been recognized to be sites for the intercellular entry of fluids (Casley Smith, 1965; Trzewik et al., 2001; Schmid-Schonbein, 2003). A transcellular fluid transport mechanism has been also reported (Lim et al., 2013; Triacca et al., 2017; Yazdani et al., 2019). When interstitial pressure increases due to local fluid accumulation and periodic tissue stress, the filaments that anchor LECs to the extracellular matrix pull on the flaps and open them, and the pressure gradients drive fluids and solutes to flow into initial lymphatics through the openings (Casley Smith, 1965; Leak, 1968; Swartz et al., 1999; Breslin, 2014). The overlapping flaps are believed to serve as primary microvalves between LECs to prevent backflow of fluids from the lymphatic lumen into the interstitial space (Schmid-Schonbein, 1990, 2003; Trzewik et al., 2001; Breslin, 2014). However, how initial lymphatics maintain their integrity while taking up fluids has been a mystery. Initial electron microscopy studies revealed that lymphatic capillaries in the ears of mice and guinea pigs possess open intercellular junctions that drained colloidal carbon, and described the existence of attachment plaques between membranes of adjacent LECs, with characteristic structural features of adherens junctions (zonula adherens) and tight junctions (zonula occludens) (Leak and Burke, 1966; Leak, 1970). A number of studies using gene profiling and cell biology approaches have also identified major junctional molecules expressed in primary and cultured LECs (Podgrabinska et al., 2002; Hirakawa et al., 2003; Johnson et al., 2006; Sironi et al., 2006; Amatschek et al., 2007; Kakei et al., 2014). The *in vivo* organization of LEC junctions, however, was unknown until high-resolution confocal microscopy studies identified discontinuous buttons in the endothelium of initial lymphatics and continuous zippers in collecting lymphatics (Baluk et al., 2007; Dejana et al., 2009a; Yao et al., 2012).

Buttons are discontinuous junctions at the sides of interdigitated membrane flaps of neighboring endothelial cells of initial lymphatics. They are enriched with adherens junction- and tight junction-associated proteins and establish anchorage points for adjoining LECs, with an orientation parallel to the cell borders. Between buttons, the flap tips overlap loosely, thereby enabling unimpeded drainage into initial lymphatics driven by interstitial-to-intraluminal pressure gradients (Baluk et al., 2007). This unique button structural organization allows for fluid entry via the tips of the LEC flaps, without the need for LEC junction dissolution, thus maintaining vessel integrity. Current evidence also supports that immune cell transmigration into initial lymphatics relies on the same button-flap structures (Pflicke and Sixt, 2009; Johnson et al., 2017), although this process is actively controlled by lymphatic endothelial chemokines and their corresponding receptors on leukocytes (Jackson, 2019b). Similar specialized buttons are observed in the initial lymphatics of many body tissues, including the trachea, dermis, diaphragm, bladder and small intestine (Baluk et al., 2007; Dejana et al., 2009a; Yao et al., 2012; Bernier-Latmani et al., 2015; Hagerling et al., 2018; Zhang et al., 2018).

It should be noted that discontinuous junctions can also occur in BECs, in response to growth factors and inflammatory mediators. However, these hyperpermeable junctions are structurally and functionally different from the buttons of initial lymphatics (Dejana et al., 2009b; Trani and Dejana, 2015). Interestingly, airway epithelium can also form intercellular openings for plasma and leukocytes to traverse from the tissue into the lumen without disrupting the integrity of the epithelial lining (Erjefalt et al., 1995; Fischer and Widdicombe, 2006).

The larger collecting lymphatic vessels are specialized for lymph transport, thus leakage should be restricted in those structures. The endothelial cells lining collecting lymphatics therefore have continuous junctions (zippers) without openings at the borders of adjacent LECs, which are similar in appearance to those seen in the endothelium of blood vessels (Baluk et al., 2007). This feature, together with the continuous basement membrane of the LECs, endows collecting lymphatic vessels with low permeability that limits lymph leakage. In addition, LECs lining lymphatic valves appear to be joined by zippers as well (Wang et al., 2016).

## Junctional Proteins in Lymphatics

Despite the striking differences in their morphology, buttons and zippers are composed of the same junctional proteins, including the adherens junction components VE-cadherin, β-catenin and p120-catenin, the tight junction components occludin, claudin-5 and ZO-1, and tight junction-associated JAM-A and ESAM (Baluk et al., 2007; Dejana et al., 2009a; Yao et al., 2012). These proteins are also expressed by BECs, where they participate in adherens and tight junction complexes at cell-cell contacts. LECs seem to organize these proteins into adherens and tight junction complexes at junctional points as well, as suggested by the ultrastructure of adherens junction and tight junction in electron microscopic images of lymphatic capillaries (Leak and Burke, 1966; Leak, 1970). In mature initial lymphatics, PECAM-1 and LYVE-1 are concentrated at the tips of LEC flaps and are spatially segregated from VE-cadherin-containing buttons at LEC borders (Baluk et al., 2007; Yao et al., 2012). Some connexins, which are components of gap junctions, are also expressed in lymphatics (Kanady et al., 2011; Meens et al., 2014, 2017; Geng et al., 2016; Munger et al., 2016, 2017; Castorena-Gonzalez et al., 2018). The expression pattern and function of these junctional proteins in the lymphatic vasculature are discussed below. Highlighted in particular are *in vivo* results documented by recent knockout mouse studies (Table 1).

**TABLE 1:** Lymphatic vascular phenotypes after genetic deletion of junctional proteins.

Genes	Mouse models	Phenotypes
**Adherens junction proteins**		
VE-cadherin (Cdh5)	*Cdh5^–/–^*	Lethality at ∼E9.5−E10.5 due to undeveloped vascular structures (Carmeliet et al., 1999; Gory-Faure et al., 1999)
	*Cdh5^f/f^;Tie2Cre*	Lethality at ∼E10.5 due to undeveloped vascular structures (Yang et al., 2019)
	*Cdh5^f/f^;Lyve-1Cre*	Lethality at ∼E10.5 probably due to defects in cardiovascular development (Yang et al., 2019)
	*Cdh5^f/f^;Prox1CreER^T2^*	Deletion at E10.5/E11.5/E12.5: severe edema and lymphatic hyperplasia at E14.5, and lethality at E14.5−E18.5. Deletion at P2/P4 and analysis at 6 week: hyperplasic and fragmentized mesenteric lymphatics and lacteals, deteriorated lymphatic valves, mildly dilated dermal initial lymphatics with button junctions maintained, unregulated expression of tight junction proteins. Adult deletion: hyperplasia and disintegration of mesenteric lymphatics and lacteals, lymphatic valve deterioration, unaltered structure of dermal initial lymphatics (Hagerling et al., 2018)
	*Cdh5^f/f^;Prox1CreER^T2^;Prox1-GFP*	Deletion at E10.5: edema and loss of lymphovenous valve formation at E16.5. Deletion at E14.5: loss of lymphatic valve formation at E18.5. Deletion at P1/P3 and analysis at P14: chylous ascites, regression of lymphatic valves (Yang et al., 2019)
β-catenin (Ctnnb1)	*Ctnnb1^f/f^;Lyve-1Cre*	Severe edema; Dilated lymphatic vessels with reduced sprouting capacity; Loss or impaired lymphovenous valve and lymphatic valve formation; Lethality before birth (Cha et al., 2016)
**Tight junction proteins**		
Claudin-5	*Claudin-5^–/–^*	Size-selective impairment of blood-brain barrier; Lethality within 10 h of birth; Lymphatic vascular phenotypes were not reported (Nitta et al., 2003)
	*Claudin-5^+/–^*	Dilated and leaky lymphatic vessels and exacerbated edema and inflammation following ultraviolet B exposure (Matsumoto-Okazaki et al., 2012)
JAM-A	*JAM-A^–/–^*	Differentially altered leukocyte trafficking (Cera et al., 2004, 2009; Woodfin et al., 2007)
	*JAM-A^f/f^;Tie2Cre*	Unaltered dendritic cell migration across lymphatic endothelium (Cera et al., 2004)
**Other junction-related proteins**		
PECAM-1	*Pecam1^–/–^*	Partially dilated and mis-branched mesenteric lymphatics and abnormal lymphatic valve formation; Unaltered leukocyte *trans*-lymphatic migration (Baluk et al., 2007; Wang et al., 2016)
	*Pecam1^–/–^;Syndecan4^–/–^*	Increased mural cell coverage and more severe lymphatic abnormalities than *Pecam1^–/–^* single mutants, which cause blood-filled lymphatic and partial lethality before birth (Wang et al., 2016)
LYVE-1	*Lyve-1^–/–^*	Delayed lymphatic trafficking of dendritic cells; Unaffected lymphatic development and drainage function (Gale et al., 2007; Luong et al., 2009; Torzicky et al., 2012; Vieira et al., 2018)
Connexins	*Connexin37^–/–^*	Lymphatic reflux; Enlargement of the jugular lymph sac; Defective lymphovenous valve and lymphatic valve formation; Unaffected lymphatic contractile capacity (Kanady et al., 2011; Sabine et al., 2012, 2015; Geng et al., 2016; Munger et al., 2016; Castorena-Gonzalez et al., 2018)
	*Connexin43^–/–^*	No mesenteric lymphatic valves; Abnormally patterned thoracic duct; Perinatal lethality due to heart defects (Kanady et al., 2011; Munger et al., 2016)
	*Connexin43^f/f^;Lyve-1Cre*	Often sudden lethal chylothorax; Impaired lymphatic valve formation; Leaky or disrupted thoracic duct; Increased lymphatic capillary branching; Unaltered lymphatic contractile capacity (Munger et al., 2017; Castorena-Gonzalez et al., 2018)
	*Connexin47^–/–^*	Normal lymphatic development and function (Munger et al., 2016; Meens et al., 2017; Castorena-Gonzalez et al., 2018)
	*Connexin37^–/–^;Connexin43^–/–^*	Severe lymphedema; Blood-filled lymphatics; Abnormal thoracic duct development; Dilated jugular lymph sac and dermal lymphatics; Perinatal lethality (Kanady et al., 2011)
	*Connexin37^–/–^;Connexin43^+/–^*	Lymphatic reflux and life-threatening chylothorax (Kanady et al., 2011)
	*Connexin43^–/–^;Connexin47^–/–^*	Mild edema; Loss of lymphovenous valve and mesenteric lymphatic valve formation; Lethality around birth (Munger et al., 2016)
	*Connexin45^f/f^;NestinCre*	Impaired entrainment of spontaneous lymphatic contraction (Castorena-Gonzalez et al., 2018)
	*Connexin45^f/f^;SmmhcER^T2^*	Reduced lymphatic contraction capacity 6–11 days after gene deletion at adult age (Castorena-Gonzalez et al., 2018)
	*Connexin26^f/f^;Keratin5Cre*	Lymphedema; Dilated vessel diameters and reduced networking in dermal lymphatics; Lethality at E16.5−E18.5 (Dicke et al., 2011)

### Adherens Junction Proteins

In blood endothelial adherens junctions, VE-cadherin mediates adhesion through homophilic interactions *in trans* and associations with an intracellular protein network including α-, β-, and γ- and p120-catenin *in cis*. The intracellular proteins β- and p120-catenin stabilize endothelial adhesion by linking VE-cadherin to the actin cytoskeleton (Dejana et al., 2009b; Dorland and Huveneers, 2017; Szymborska and Gerhardt, 2018). In the lymphatic endothelium on the other hand, VE-cadherin is present at all junction types, and its distribution is particularly enriched in buttons (Baluk et al., 2007; Dejana et al., 2009a; Yao et al., 2012). β- and p120-catenin colocalize with VE-cadherin at both buttons and zippers between LECs (Yao et al., 2012), but how adherens junction complexes are organized at these junctions is unclear. In BECs, VE-cadherin phosphorylation, internalization and degradation in response to differential vascular cues regulate the assembly and stability of adherens junctions and vascular permeability (Dejana et al., 2009a; Orsenigo et al., 2012; Conway et al., 2017). Whether these mechanisms are responsible for button and zipper junction organization in LECs remains to be further investigated.

Constitutive loss of VE-cadherin causes embryonic lethality at approximately embryonic day (E) 9.5−E10.5, before the development of lymphatic vasculature (Carmeliet et al., 1999; Gory-Faure et al., 1999; Yang et al., 2019). Studies using tamoxifen-inducible genetic models have revealed that prenatal LEC-specific deletion of VE-cadherin leads to an aberrant primitive lymphatic plexus, loss of lymphatic valves, edema and fetal death (Hagerling et al., 2018; Yang et al., 2019). Similarly, mice with constitutive deletion of β-catenin in LECs exhibit significantly dilated lymphatic sacs, lack of lymphatic valves, severe edema and prenatal lethality (Cha et al., 2016). Postnatal or adult deletion of VE-cadherin also impairs the integrity of lacteals and mesenteric lymphatic collectors and the maintenance of lymphatic valves, whereas mature adult dermal lymphatics are resistant to VE-cadherin loss (Hagerling et al., 2018; Yang et al., 2019). Interestingly, VE-cadherin loss causes chylous ascites, possibly resulting from lymph leakage from mesenteric collecting vessels due to endothelial barrier dysfunction, as well as valve deterioration, which enables retrograde flow of lymph from larger collecting vessels into lymphatic tributaries where chyle is prone to leak (Nitschke et al., 2017). Growing *in vivo* evidence (Hagerling et al., 2018; Yang et al., 2019) supports that the lymphatic vasculature uses VE-cadherin as a mechanotransducer at junctional sites, similarly to the blood vasculature (Tzima et al., 2005; Coon et al., 2015). Due to the high flow shear at valve sinuses, lymphatic valves may be particularly susceptible to defective cell-cell adhesion and mechanotransduction; this could explain why lymphatic valves deteriorate upon VE-cadherin deletion. At the molecular level, VE-cadherin regulates a number of signaling pathways that are crucial for LEC junction maturation, proliferation and mechanotransduction, including the β-catenin, VEGFR/AKT, YAP/TAZ, PROX1 and FOXC2 pathways (Sabine et al., 2015; Hagerling et al., 2018; Yang et al., 2019). Taken together, these results suggest that VE-cadherin, in addition to mediating cell-cell adhesion, acts also as a membrane signaling hub to transfer intracellular signals in LECs.

### Tight Junction Proteins

Tight junctions join endothelial cells of all blood vessels and are most abundant in cerebrovascular endothelial cells. In brain microvessels, multiple interconnected strands of tight junction structures are present at the apical borders of joining endothelial cells, thereby limiting permeability of the blood-brain barrier (Stamatovic et al., 2008; Dejana et al., 2009b; Greene et al., 2019). As mentioned previously, in LECs both buttons and zippers express the endothelial tight junction constituents claudin-5, occludin and ZO-1, as well as the associated proteins JAM-A and ESAM. Although the distribution of those proteins overlaps with VE-cadherin (Baluk et al., 2007; Dejana et al., 2009a; Yao et al., 2012), VE-cadherin deletion or functional inactivation does not alter the distribution of tight junction proteins in buttons and zippers, indicating that tight junction organization is independent of adherens junctions in lymphatic vessels (Baluk et al., 2007; Hagerling et al., 2018). As previously shown in epithelial cells and BECs, tight junctions usually occupy the apical end of the junctional clefts, while adherens junctions are more basal (Niessen, 2007; Dejana et al., 2009a). It is possible that this organization is also maintained at buttons and zippers in LECs. The expression of tight junction proteins such as claudin-5, ZO-1 and JAM-A is upregulated in VE-cadherin-deleted LECs, which may represent a compensatory response following the loss of VE-cadherin (Hagerling et al., 2018). Claudin-5 is a key constituent of endothelial tight junctions. *Claudin-5^–/–^* mice die within 10 h of birth, and their blood-brain barrier function is impaired against small molecules (Nitta et al., 2003). Importantly, *Claudin-5^+/–^* mice show dilated and leaky lymphatics after ultraviolet B irradiation (Matsumoto-Okazaki et al., 2012). While the exact roles of claudin-5 in different lymphatics have yet to be elucidated, these results indicate that a claudin-5-dependent barrier may be required for the maintenance of collective lymphatic vessel integrity. Finally, the expression of the tight junction-associated protein JAM-A in LECs does not play a role in lymphatic regulation of leukocyte trafficking (Cera et al., 2004), despite the fact that JAM-A expressed in BECs and certain leukocyte populations, such as dendritic cells, neutrophils and monocytes, has been shown to facilitate leukocyte transmigration (Cera et al., 2004; Woodfin et al., 2007; Cera et al., 2009; Schmitt et al., 2014). Taken together, our understanding of lymphatic tight junctions is still limited, and the dynamics of tight junction proteins in lymphatic development and function have yet to be elucidated.

### PECAM-1

The endothelial adhesion molecule PECAM-1 is expressed at a high density at the cell borders of all endothelial cells in the blood and lymphatic vasculature (Sauter et al., 1998; Torzicky et al., 2012). In blood vessels, PECAM-1 acts as a regulator of endothelial cell junction integrity and mechanotransduction and plays an important role in leukocyte transendothelial migration (Conway et al., 2013; Privratsky and Newman, 2014; Chistiakov et al., 2016). PECAM-1 distribution in LECs shows partial overlap with VE-cadherin in buttons and zippers, but it does not appear to regulate VE-cadherin localization, or the junctional integrity of buttons (Baluk et al., 2007). In lymphatic capillaries, PECAM-1 lines mostly the tips of flaps that constitute channels of leukocyte entry (Baluk et al., 2007). To this end, LECs express the chemokine CCL21, which is sensed by the leukocyte receptor CCR7 and provides migration cues for skin dendritic cells toward initial lymphatics (Forster et al., 1999; Saeki et al., 1999). *In vitro*, a PECAM-1-blocking antibody inhibits dendritic cell migration across TNFα-inflamed LEC monolayers and skin explants, suggesting that PECAM-1 may regulate leukocyte migration into lymphatics (Torzicky et al., 2012). However, such a role is not supported by *in vivo* evidence, as *Pecam1-null* mice have normal leukocyte trafficking into afferent lymphatics (Baluk et al., 2007). Finally it has been suggested that PECAM-1 may act as a regulator of lymphatic valve formation and a flow sensor in LECs (Wang et al., 2016).

### LYVE-1

Lymphatic vessel endothelial receptor 1 is the major receptor for the ubiquitous glycosaminoglycan hyaluronan in the lymphatic vasculature (Banerji et al., 1999) with a preferential distribution in initial lymphatics over large collective vessels (Baluk et al., 2007), although it is also expressed in some macrophage and BEC populations (Mouta Carreira et al., 2001; Cho et al., 2007; Xu et al., 2007; Gordon et al., 2008; Lim et al., 2018). *Lyve-1-null* mice exhibit normal lymphatic development and tissue fluid drainage, suggesting that LYVE-1 is not required for these lymphatic functions (Gale et al., 2007; Luong et al., 2009). However a role for LYVE-1 as a lymphatic-specific receptor for leukocyte trafficking has been recently demonstrated (Lawrance et al., 2016; Johnson et al., 2017; Vieira et al., 2018; Jackson, 2019a). Dendritic cells express hyaluronan on their surface, which during *trans*-lymphatic migration binds to LYVE-1-lined ring-like openings at LEC borders (Johnson et al., 2017). This interaction enables dendritic cell docking to the basolateral surface of initial lymphatics, and the openings allow for dendritic cell entry (Johnson et al., 2017; Jackson, 2019a). In addition to dendritic cells, macrophages may also utilize the same LYVE-1-hyaluronan axis for transport through inflamed lymphatic endothelium (Lawrance et al., 2016; Vieira et al., 2018). LYVE-1 internalization (Johnson et al., 2007) or shedding (Wong et al., 2016) from the lymphatic endothelium allows for the subsequent release of leukocytes into the lymphatic vessel lumen. These findings illustrate a role for the LYVE-1-hyaluronan complex in leukocyte trafficking through initial lymphatic openings defined by button junctions. Notably, previous studies have revealed that in inflamed tissues, dendritic cell trafficking into lymphatics requires β1- and β2-integrins on dendritic cells to engage with their counter receptors ICAM-1 and VCAM-2 on LECs (Johnson et al., 2006; Teijeira et al., 2013; Jackson, 2019a). It has been hypothesized that the LYVE-1-hyaluronan and integrin-ICAM-1/VCAM-2 pathways may coordinate the stepwise entry of leukocytes into afferent lymphatics (Jackson, 2019a). However, this stands in contrast to the observations that lymphatic transmigration and many other inflammatory responses remain unaffected in *LYVE-1-null* mice (Gale et al., 2007; Luong et al., 2009). One possibility is that other lymphatic receptors could compensate for the loss of LYVE-1 function during leukocyte transmigration. Further studies to identify such factors and the possible underlying mechanisms are warranted.

### Connexins

Connexins are a family of transmembrane proteins consisting of 21 members in human and 20 members in mouse that assemble into pore-forming hexamers called connexons. Connexons can function as hemichannels in the plasma membrane for cytosol-extracellular diffusion or dock together across adjacent cells to form complete intercellular gap junctions that mediate direct cell-cell communication (Goodenough and Paul, 2009). The role of connexin-mediated homocellular or heterocellular coupling of BECs and vascular smooth muscle cells has been well studied in blood vessels (Figueroa and Duling, 2009; Okamoto and Suzuki, 2017; Okamoto et al., 2019). In contrast, much less is known about connexins in lymphatic vessels; their *in vivo* expression pattern and function in LECs and lymphatic smooth muscle cells have only begun to be unveiled in recent years.

Kanady et al. (2011) showed that Connexin37, Connexin43, and Connexin47 are variably expressed in the endothelium of developing and mature collecting lymphatics. Both Connexin37 and Connexin43 are crucial for normal lymphatic development and function, as evident by the various abnormalities in lymphatic vessel patterning and valve formation, and the presence of lymphedema in *Connexin37*-, *Connexin43-* or *Connexin37*;*Connexin43-null* mice (Kanady et al., 2011; Sabine et al., 2012, 2015; Munger et al., 2016, 2017; Table 1). During embryogenesis, Connexin37 is required for maintaining the proper size of the jugular lymph sac, and both Connexin37 and Connexin43 critically regulate thoracic duct development. Intriguingly, Connexin43 and Connexin37 are enriched in a subset of LECs in mature lymphatic valves and have a differential distribution at the upstream and downstream sides of the valves, possibly due to unequal flow shear forces exerted on either side of the valve (Kanady et al., 2011). High expression of Connexin37 in valve LECs depends on PROX1, FOXC2 and flow shear stress (Sabine et al., 2012, 2015). Consistent with their abundant expression in valve LECs, deletion of *Connexin37*, *Connexin43* or both, results in partial or complete loss of lymphatic valve formation (Kanady et al., 2011; Sabine et al., 2012, 2015; Munger et al., 2016, 2017). On the other hand, connexin47 expression is highly restricted to a very small portion of valve LECs on the upstream side of the valve, but the ablation of *Connexin47* alone does not result in lymphatic valve defects or lymphedema in mice (Munger et al., 2016; Meens et al., 2017). Interestingly, mutations in *GJA1* (Connexin43) and *GJC2* (Connexin47) have been linked to human primary and secondary lymphedema (Ferrell et al., 2010; Ostergaard et al., 2011; Finegold et al., 2012; Brice et al., 2013). However, Connexin43 or Connexin47 single mutant mice exhibit no lymphedema, and the lymphedema phenotype can only been seen in *Connexin37^–/–^;Connexin43^–/–^* mice and some *Connexin43^–/–^;Connexin47^–/–^* mutant mouse embryos (Kanady et al., 2011; Sabine et al., 2012). Furthermore, human patients with connexin mutations show no signs of the developmental lymphatic valve abnormalities observed in *Connexin47*-, *Connexin43-* or *Connexin37-null* mice (Ferrell et al., 2010; Ostergaard et al., 2011; Finegold et al., 2012; Brice et al., 2013). These discrepancies raise the question of how the mechanism underlying the defective lymphatic vessel and valve development in connexin mutant mice could relate to the pathogenesis of lymphedema in human patients carrying connexin mutations.

The smooth muscle cell layer of collecting lymphatics generates intrinsic spontaneous contractions that are required for propulsion of lymph. Therefore, loss of lymphatic contractile capacity due to smooth muscle cell dysfunction may be a component of lymphedema and other diseases that are characterized by deficient lymphatic transport. Connexin45, the predominant connexin isoform in lymphatic smooth muscle cells, is essential to the entrainment of lymphatic contraction waves. Connexin45 deficiency disrupts the electrical communication in the smooth muscle layer of lymphatics and largely impairs lymphatic contraction and pumping capability (Castorena-Gonzalez et al., 2018). However, these defects do not result in inhibition of lymph transport *in vivo*, unless a chronic gravitational load is imposed. This is consistent with evidence that lymph transport relies not only on smooth muscle contraction, but also on other driving forces (e.g., interstitial pressure). A partial-to-severe loss of lymphatic pumping function alone is insufficient to abolish forward movement of lymph. Nevertheless, *GJC1* (Connexin45) mutations have not been identified in human lymphedema patients (Srinivas et al., 2018). Thus, whether a Connexin45 deficiency in lymphatic smooth muscle cells is involved in the pathogenesis of this disease remains yet unclear.

Despite the important advances in understanding the roles of connexins in the lymphatic vasculature, many fundamental questions about lymphatic connexins remain unanswered. First and most importantly, are connexins assembled into gap junction structures in adjacent LECs and smooth muscle cells in the lymphatic wall? If yes, are they integrated with other junctional proteins? In which signaling pathways are connexins involved in those cells? Finally, elucidating the interplay between LEC and smooth muscle cell connexins in mediating cytosol-to-extracellular and cell-to-cell communications will be of particular importance in order to understand key lymphatic functions, such as lymphatic vessel growth, contraction and flow responses.

## LEC Junctions in Development

In mice, the development of the lymphatic vasculature is initiated at approximately E9.5, when a population of endothelial cells in the anterior cardinal vein start expressing PROX1 and become lymphatic endothelial progenitor cells (Wigle and Oliver, 1999; Srinivasan et al., 2007; Escobedo and Oliver, 2016). The VEGF-C/VEGFR3 signaling axis subsequently drives budding of PROX1-positive cells from the cardinal vein to form primitive lymphatic structures called lymph sacs (Karkkainen et al., 2004; Hagerling et al., 2013). As developmental lymphangiogenesis proceeds, new lymphatic vessels grow out centrifugally of the lymph sacs and eventually form the majority of the lymphatic vascular tree (Vaahtomeri et al., 2017). Of note, recent lineage-tracing studies (Klotz et al., 2015; Martinez-Corral et al., 2015; Stanczuk et al., 2015) have identified additional LEC progenitors from non-venous origins (e.g., hemogenic endothelium) in multiple organs including skin dermis, heart and mesentery that also give rise to new lymphatics. The precise cell identity of these precursors deserves further studies.

Interestingly, developing lymphatics have zippers, but not buttons. Yao et al. (2012) showed that LECs in the E12.5 jugular lymph sacs and E16.5 trachea lymphatics are joined exclusively by zipper junctions. Importantly, LEC progenitors budding from the cardinal vein at E10.5−E12.5 are also connected to each other by zippers (Yang et al., 2012; Yao et al., 2012). Similar junctions are present at the tips of growing lymphatic sprouts regardless of age (Baluk et al., 2007; Yang et al., 2012; Yao et al., 2012). Hence, when lymphatic budding or sprouting takes place, zipper junctions are developed to maintain close cell-cell contacts between LECs in such a manner that these cells can migrate as a cohesive unit. This is in agreement with the general requirement for tight cell-cell junctions during collective cell movement, in order to maintain tissue integrity, control cell polarity and ensure cell-cell communication (Ilina and Friedl, 2009; Friedl and Mayor, 2017). Indeed, targeted disruption of VE-cadherin in LECs starting at E10.5 prevents primitive lymphatic structures from remodeling and maturing, supporting the essential role of stable zipper junctions during lymphatic development (Hagerling et al., 2018). On the other hand, excessive stabilization of endothelial cell junctions in mice due to covalent fusion between VE-cadherin and α-catenin strongly suppresses embryonic lymphangiogenesis (Dartsch et al., 2014), suggesting that lymphatic development critically depends on the balanced adhesive strength of zipper junctions. PROX1 (Johnson et al., 2008), RASIP1 (Liu et al., 2018), GATA2 (Mahamud et al., 2019) and miR-126 (Mahamud et al., 2019) have been identified as important regulators of VE-cadherin as well as other junction proteins in developing lymphatics. In conclusion, several lines of evidence support that VE-cadherin dynamics at adherens junctions of zippers are very important for lymphatic vessel development (Baluk et al., 2007; Yang et al., 2012, 2019; Yao et al., 2012; Hagerling et al., 2018). Whether tight junction and gap junction proteins contribute to the formation of zipper junctions and collective cell migration in developing lymphatics is currently unknown.

Buttons start to replace zippers in initial lymphatics at E16.5−E17.5 (Yao et al., 2012; Zheng et al., 2014). As lymphatic capillary networks develop, buttons become more abundant from E17.5 to birth and into adulthood and eventually occupy almost 100% of junctional points in the lymphatic capillaries at postnatal day 70. Intermediates of the two junction types appear during the process of button formation (Yao et al., 2012). At the same time, junctions in the endothelium of collecting lymphatic vessels remain zippers (Yao et al., 2012; Zheng et al., 2014). Taken together, the facts that (1) the appearance and disappearance of intermediate junctions coincide with a decrease in zippers and increase in buttons, and (2) all of these junction types have the same protein composition strongly suggest that zippers in developing initial lymphatics transform into buttons via intermediate forms. This process, referred to as “zipper-to-button” transformation, may involve redistribution of existing junctional structures at cell borders rather than assembly of new ones. The precise molecular mechanisms responsible for button formation during development remain largely unknown. It has been postulated that birth may induce rapid button formation in order to guarantee efficient clearance of amniotic fluid by lung lymphatics after the onset of breathing and immediate absorption of dietary fat by lacteals after initiation of feeding (Kulkarni et al., 2011; Yao et al., 2012; Zhang et al., 2018). The emergence of buttons is coincident with the establishment of a fully functional immune system, raising the question of whether developing immune cells are involved in the regulation of button formation and maturation. Previous studies have suggested that dexamethasone and the growth factor angiopoietin-2 (ANGPT2) promote button formation during development (Yao et al., 2012; Zheng et al., 2014), whereas inflammation or inhibition of a few endothelial factors leads to a reversal process, “button-to-zipper” transformation (Yao et al., 2012; Bernier-Latmani et al., 2015; Zhang et al., 2018). These findings will be discussed subsequently.

## LEC Junctions in Lipid Uptake and Obesity

One of the main functions of lymphatic vessels of intestinal villi (lacteals) is to absorb dietary lipids, or chylomicrons, from the gut and transport them to the blood. The intestinal lymphatic system consists of lacteals, which reside at the center of intestinal villi and take up chylomicrons, and mesenteric collectors that drain chylomicrons into the thoracic duct and eventually into the venous circulation (Randolph and Miller, 2014; Bernier-Latmani and Petrova, 2017; Petrova and Koh, 2018; Cifarelli and Eichmann, 2019). Despite the recognized importance of lacteals as mediators of lipid uptake, the cellular mechanisms controlling chylomicron entry into lacteals have remained elusive for years. Some reports have suggested that chylomicrons are preferentially taken up by a specialized subset of cells located at the lacteal tip (Lee, 1986; Randolph and Miller, 2014), while studies based on electron microscopy have suggested that chylomicrons enter through LEC junctions in lacteals or pass through lacteals by transcytosis (Dixon, 2010a, b). We and others have shown that under baseline conditions, lacteals display predominately buttons (Bernier-Latmani et al., 2015; Zhang et al., 2018), thus demonstrating features of initial lymphatics. Ultrastructural visualization of wild-type mouse intestinal villi has clearly shown that chylomicrons enter the lacteal lumen through junctional openings on the lacteal wall (Palay and Karlin, 1959; Casley-Smith, 1962; Zhang et al., 2018). Indeed, lipid absorption by lacteals requires the presence of button junctions. Certain endothelial signaling pathways, such as VEGF-A/VEGFR2, DLL4/Notch and ROCK signaling, critically control lipid uptake by regulating lacteal junction morphology (Bernier-Latmani et al., 2015; Zhang et al., 2018, see below). Most importantly, inducing button-to-zipper transformation by targeting the aforementioned signaling pathways, prevents lipid uptake into the bloodstream, thereby rendering mice resistant to diet-induced obesity and associated metabolic syndromes. Similarly, microbiota depletion in the small intestine has been shown to compromise lacteal maturation and maintenance and induce lacteal junction zippering, leading to impaired lipid absorption (Suh et al., 2019). Thus, gut microbiota is also essential for maintaining lacteal button junctions, and plays a crucial role in lipid uptake in the small intestine (Pascale et al., 2019). As such, gut microbiota might be a suitable therapeutic target against obesity and other metabolic disorders. Collectively, these findings suggest that chylomicron uptake by lymphatics relies on lacteal button junctions, and that zippering (closing) of the lacteal junctions represents an attractive novel strategy for preventing plasma lipid uptake and obesity.

*Prox1^+/–^* mice, which present dilated and leaky mesenteric lymphatics possibly due to lymphatic cell junction defects, exhibit excessive accumulation of perimesenteric fat and adult-onset obesity (Harvey et al., 2005; Johnson et al., 2008; Escobedo et al., 2016). In line with this, *Apelin-null* mice fed with a high fat diet for 5 weeks develop a more obese phenotype, which is attributed to impaired lymphatic and blood vessel integrity and increased leakiness during lymph transport (Sawane et al., 2013). Taken together, it appears that loss of junction integrity on collecting lymphatics leads to chronic lymph leakage and can trigger late-onset obesity. Thus, tightening junctions in lymphatic collectors may also be useful in order to prevent or treat adult obesity.

## LEC Junctions in Inflammation-Associated Conditions

A number of inflammatory diseases, such as respiratory tract inflammation and inflammatory bowel disease, are accompanied by lymphangiogenesis (Kim et al., 2014). Detailed characterization of trachea lymphatics after *Mycoplasma pulmonis* infection suggests that such newly formed inflammation-induced lymphatics have predominantly zippers (Baluk et al., 2007; Yao et al., 2012). This is in agreement with the idea that zippers are a general feature of all growing lymphatics under physiological and pathological conditions. Notably, buttons in existing lymphatics also transform into zippers during sustained inflammation and then gradually revert to buttons upon inflammation resolution (Yao et al., 2012), but the molecular basis of inflammation-induced LEC junction remodeling is still largely unknown. Functionally, junction zippering would make the inflamed lymphatics less permeable, thereby reducing fluid entry and clearance and aggravating tissue edema. In support of this, lymph flow is decreased during chronic inflammation (Huggenberger et al., 2010; Zhou et al., 2010; Cromer et al., 2015), although increased lymph flow may take place at the onset of acute inflammation (Benoit et al., 1989; Zhou et al., 2010). As described previously, dendritic cells also utilize lymphatic button junctions; thus junction zippering may be detrimental for dendritic cell trafficking into lymphatics. Yet, enhancement of lymphatic trafficking of dendritic cells has been frequently described in the context of inflammation (MartIn-Fontecha et al., 2003; Johnson et al., 2006; Vigl et al., 2011; Russo et al., 2013). This raises the possibility that leukocytes can migrate across zipper junctions in lymphatic vessels under inflammatory conditions, similarly to what has been described for blood vessels (Muller, 2015). Clearly, more research in this area is required to understand how inflammation-induced alterations in lymphatic junctions affect immune cell trafficking and resolution of tissue inflammation.

Notably, inflammation and infections have been implicated in the pathogenesis of secondary lymphedema (Yuan et al., 2019). Although how exactly inflammation alters lymphatics structurally and functionally in the context of lymphedema remains to be determined, it is possible that junction zippering, along with other potential lymphatic dysfunctions such as retrograde lymph flow, underlies or contributes to localized fluid retention and tissue swelling in patients with inflammation-associated lymphedema. If this hypothesis is true, promoting button formation may improve lymphatic drainage function and reduce edema in these patients, hereby representing a novel therapeutic approach against this, notoriously difficult to treat, disease.

## Molecular Regulation of LEC Junctions

### ANGPT2

Angiopoietin-2 is a multifaceted growth factor that regulates blood and lymphatic vascular functions. In the blood vasculature, it can function as an agonist or an antagonist of ANGPT1/TIE2 pathway in different contexts (Kim et al., 2016; Korhonen et al., 2016). In the lymphatic endothelium, ANGPT2 promotes embryonic and postnatal lymphangiogenesis by acting primarily as an agonist of the TIE2 receptor (Gale et al., 2002; Dellinger et al., 2008; Souma et al., 2018). LECs express low levels of the orphan receptor TIE1 and no VE-PTP, which catalyzes the dephosphorylation of TIE2. This expression pattern is thought to contribute to the preferential role of ANGPT2 as a TIE2 agonist in lymphatics, given that ANGPT2-mediated TIE2 antagonism is largely dependent on TIE1/TIE2 interaction and VE-PTP activity (Song et al., 2012; Souma et al., 2018). Additional information on the context-dependent roles of ANGPT2 in angiogenesis and lymphangiogenesis is reviewed in detail by Akwii et al. (2019).

Beyond these activities, ANGPT2 has also been identified as an essential regulator of LEC junctions in developing lymphatic vessels. Inhibition of ANGPT2 prevents transformation of junctions from zippers to buttons in initial lymphatics during late gestation (Zheng et al., 2014), suggesting a key role of ANGPT2 in promoting junction maturation. Consistent with this, loss of ANGPT2 function leads to compromised drainage capacity and edema (Dellinger et al., 2008; Zheng et al., 2014). Interestingly, ANGPT2 inhibition also concomitantly reduces VE-cadherin phosphorylation at tyrosine residue 685 in developing initial lymphatics (Zheng et al., 2014), raising the possibility that ANGPT2 promotes button formation by controlling the phosphorylation state of VE-cadherin. In addition, ANGPT2 blockage or deletion also largely impairs junction integrity and valve formation in collecting lymphatics before and after birth, thereby resulting in lymph leakage and chylous ascites (Dellinger et al., 2008; Zheng et al., 2014). In summary, ANGPT2 is required for proper patterning of both button and zipper junctions in lymphatics during development. However, the involvement of ANGPT1 and TIE2 receptor in this process has yet to be determined.

### Dexamethasone

Dexamethasone is widely used to treat inflammation by acting as an agonist of glucocorticoid receptor. Treatment with dexamethasone has been found not only to reduce VEGF-C-induced lymphangiogenesis, but also to induce the transformation of zippers into buttons in inflamed initial lymphatics (Yao et al., 2010, 2012). This supports the idea that button formation is beneficial to inflammation resolution. Dexamethasone also promotes button formation during neonatal stages in the absence of inflammation (Yao et al., 2012; Zheng et al., 2014), suggesting a direct effect of the steroid on lymphatic junction morphology. The signaling components that mediate the effects of dexamethasone on LEC junctions are not known. As glucocorticoid receptor activation in LECs occurred concomitantly with the formation of button junctions after treatment with dexamethasone (Yao et al., 2012), the junction remodeling is possibly mediated by glucocorticoid receptor signaling. Intriguingly, a number of studies have shown that dexamethasone enhances junctional integrity of BECs by stabilizing the anchorage of adherens junction and tight junction proteins to actin filaments and maintaining the levels of junctional components, thereby reducing vascular permeability under pro-inflammatory conditions (Romero et al., 2003; Blecharz et al., 2008; Salvador et al., 2014). Taken together, these results reveal an interesting context-dependent action of dexamethasone in the regulation of LEC and BEC junctions.

### VEGF-A/VEGFR2 Signaling

Vascular endothelial growth factor-A is a major angiogenic growth factor. It signals by binding to its primary receptor VEGFR2 expressed on the surface of BECs and LECs. VEGF-A binding induces tyrosine phosphorylation of VEGFR2 at specific sites and triggers downstream signaling events that are critical for BEC survival, proliferation and migration during angiogenesis (Eichmann and Simons, 2012; Koch and Claesson-Welsh, 2012). VEGF-A signaling through VEGFR2 in LECs is also known to promote lymphatic growth before birth, but does not appear to have important impact on postnatal lymphangiogenesis (Dellinger and Brekken, 2011; Dellinger et al., 2013; Zarkada et al., 2015). In blood endothelium, VEGF-A acts also to transiently increase vascular leak by opening cell-cell junctions (Simons et al., 2016; Dorland and Huveneers, 2017). One well studied pathway that leads to VEGF-A induced BEC junction opening involves the phosphorylation of VEGFR2 tyrosine residue, Y951 (Y949 in the mouse) and the subsequent recruitment of the adaptor protein TSAD, activation of SRC and FAK, VE-cadherin phosphorylation and internalization, and ultimate adherens junction disassembly (Matsumoto et al., 2005; Ruan and Kazlauskas, 2012; Sun et al., 2012; Li et al., 2016). Another pathway involves activation of the small GTPase RhoA and ROCK downstream of VEGFR2, which drives rearrangement of cortical actin into perpendicular stress fibers that bind to the VE-cadherin cytoplasmic tail and pull BEC junctions to open (van Nieuw Amerongen et al., 2003; Bryan et al., 2010; Di Lorenzo et al., 2013).

**FIGURE 2:**
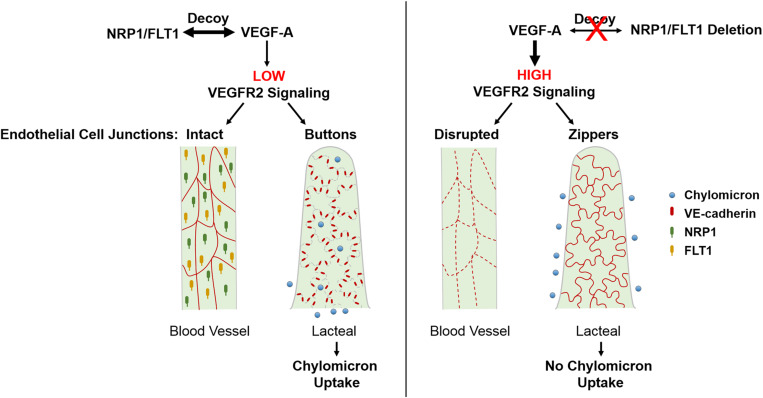
Regulation of endothelial cell-cell junctions by VEGF-A/VEGFR2 signaling in intestinal villi. VEGF-A bioavailability for VEGFR2 is limited due to VEGF-A binding to FLT1 and NRP1 which are only highly expressed in BECs. This results in continuous junctions in BECs and discontinuous buttons in LECs that allow for lacteal chylomicron uptake. Increased levels of VEGF-A or deletion of *Nrp1 and Flt1* in BECs leads to upregulated signaling through VEGFR2, which disrupts BEC junctions and, opposingly, leads to lacteal junction transformation from buttons to zippers. As a result, chylomicron uptake by lacteals is inhibited, rendering mice resistant to diet-induced obesity. BEC, blood endothelial cell; LEC, lymphatic endothelial cell; NRP1, neuropilin1; FLT1, Fms-related tyrosine kinase 1 (or VEGFR1).

In addition to VEGFR2, VEGF-A also binds to VEGFR1 (or FLT1) and NRP1 (Hiratsuka et al., 1998; Gelfand et al., 2014). In the intestine, these two receptors are not expressed by intestinal LECs, but only BECs (Jurisic et al., 2012). Previous studies suggested that FLT1 has limited intrinsic tyrosine kinase activity in endothelial cells and rather acts as a decoy that prevents VEGF-A signaling through VEGFR2 (Hiratsuka et al., 2005; Shibuya, 2006). On the other hand, NRP1 is a single-pass non-tyrosine kinase transmembrane receptor that stimulates angiogenesis and tip cell formation via VEGFR2 dependent and independent mechanisms (Fantin et al., 2013, 2015; Gelfand et al., 2014; Aspalter et al., 2015). Interestingly, NRP1 is expressed in lymphatic valves and promotes valve formation together with PlexinA1, via interactions with semaphorin3A (Bouvree et al., 2012). We have recently reported (Zhang et al., 2018) that mice with endothelial cell-specific deletions of both *Flt1* and *Nrp1* had reduced lipid absorption into plasma and were resistant to high fat diet-induced obesity. Mechanistically, endothelial deletion of *Flt1* and *Nrp1* led to enhanced VEGFR2 signaling and induced formation of zipper junctions in intestinal lacteals that inhibited chylomicron uptake. Similarly, increasing VEGFR2 signaling via VEGF-A stimulation promoted zippering of lacteal junctions. This stands in contrast to blood capillaries, where increased VEGFR2 signaling opens BEC junctions. Conversely, transient inhibition of VEGFR2 signaling in the double mutant mice could rescue button junctions and chylomicron uptake. As expected, LEC-specific deletion of *Nrp1* and *Flt1* receptors did not affect lacteal junctions or weight gain in mice on a high fat diet. Taken together, our results point to a cell non-autonomous effect on LEC junctions, mediated by NRP1 and FLT1 expressed by villus BECs. These data show that elevating VEGF-A/VEGFR2 signaling induces lacteal junction zippering, and that FLT1 and NRP1 in BECs function as VEGF-A decoys to antagonize this effect (Figure 2). Developmentally, VEGF-A/VEGFR2 signaling together with VEGF-C/VEGFR3 provides prenatal LEC growth signals (Nagy et al., 2002; Wirzenius et al., 2007; Dellinger et al., 2013). However, postnatal lacteal LECs must switch from a growth state with zipper junctions to a functional state with button junctions to allow chylomicron uptake and efficient nutrition of the newborn mouse. This may entail VEGF-A antagonization by NRP1 and FLT1, thereby allowing lacteal junction maturation and chylomicron uptake after birth. This process is likely to be initiated by activation of the chylomicron processing proteins MTP and ApoB at birth (Zhang et al., 2018), which leads to upregulation of FLT1 expression on BECs via ApoB (Avraham-Davidi et al., 2012).

Collectively, these findings identify a novel role for VEGF-A/VEGFR2 signaling in controlling LEC junction remodeling and uptake in intestinal lacteals, although the molecular basis of this effect is still unclear. Interestingly, treatment of wildtype mice with the ROCK inhibitor Y27632 promoted LEC junction zippering and inhibited chylomicron uptake into lacteals (Zhang et al., 2018). This raises the possibility that VEGFR2 signaling in LECs induces junction remodeling by inhibiting RhoA-ROCK signaling-mediated cytoskeletal rearrangement. Signaling through the VEGFR2 tyrosine residue Y949 is another pathway that may mediate VEGF-A-induced lacteal junction zippering. Evidently, further studies are necessary to elucidate the cell autonomous roles of VEGFR2 and downstream signaling pathways that regulate LEC junction remodeling. Finally it would be of interest to understand the opposing effects of VEGF-A on blood and lymphatic vasculature and to identify effectors that regulate the morphological status of lacteal junctions.

### DLL4

Delta-like 4 is a major canonical Notch ligand expressed in vascular endothelial cells. DLL4/Notch signaling interplays with many other signaling pathways, such as VEGF-A/VEGFR2 and VEGF-C/VEGFR3 pathways, and has been shown to be essential for sprouting angiogenesis and vascular differentiation under various physiological and pathological conditions (Hellstrom et al., 2007; Lobov et al., 2007; Siekmann and Lawson, 2007; Suchting et al., 2007; Benedito et al., 2012). DLL4 is also highly expressed in the lymphatic endothelium. Genetic inactivation of *Dll4* in LECs leads to lacteal regression accompanied by “button-to-zipper” transformation of lacteal junctions and reduced lipid uptake and transport capacity (Bernier-Latmani et al., 2015). This underlies, at least in part, the decreased fat accumulation and improved metabolic parameters observed in HFD-fed mice after DLL4 blockage (Fukuda et al., 2012). The cardioprotective peptide adrenomedullin and its signaling partners Calcrl and RAMP2 critically regulate lymphangiogenesis during development or after myocardial infarction (Fritz-Six et al., 2008; Jin et al., 2008; Klein and Caron, 2015; Trincot et al., 2019), while Calcrl has been identified as an important upstream regulator of DLL4-Notch signaling (Davis et al., 2017). Through controlling DLL4 expression in LECs, Calcrl-adrenomedullin signaling is essential for maintenance of proper junction organization in lacteals, thereby contributing to lipid uptake and inflammation resolution (Davis et al., 2017). As *Dll4* deletion can enhance VEGFR2 signaling (Williams et al., 2006; Suchting et al., 2007), it is possible that the “button-to-zipper” conversion phenotype in *Dll4-null* lacteals is a result of increased LEC VEGFR2 signaling, as discussed above. The exact crosstalk between Notch and VEGFR2 and its implications in LEC junction remodeling remain to be further addressed.

### VEGF-C/VEGFR3 Signaling

Vascular endothelial growth factor-C signaling through VEGFR3 is essential for lymphangiogenesis. After the first PROX1-positive LEC progenitors egress from the anterior cardinal vein in mouse embryos at ∼E10.5, VEGF-C/VEGFR3 signaling critically regulates a variety of LEC functions, such as proliferation, migration, differentiation and apoptosis, thereby contributing to prenatal and postnatal expansion and maintenance of the lymphatic vasculature (Secker and Harvey, 2015; Petrova and Koh, 2018). The VEGF-C coreceptor NRP2 modulates lymphatic vessel sprouting together with VEGFR3 (Xu et al., 2010). VEGFR3 signaling also contributes to blood vessel growth (Siekmann and Lawson, 2007; Tammela et al., 2008, 2011; Zarkada et al., 2015) and restrains vascular permeability by limiting VEGF-A/VEGFR2 signaling (Heinolainen et al., 2017). Yet little is known about the role of VEGFR3 signaling in junction dynamics in LECs. *In vitro* studies suggested that stimulation with VEGF-C or the VEGFR3 selective activator VEGF-C156S, slightly decreases transendothelial electrical resistance in cultured LEC monolayers (Breslin et al., 2007). Moreover, systemic delivery of VEGF-C via adenovirus promotes colorectal tumor-associated lymphangiogenesis, while reducing VE-cadherin expression and lymphatic endothelial barrier integrity (Tacconi et al., 2015). However, recent studies have argued that VEGF-C signaling through VEGFR3 does not affect LEC junction organization either *in vivo* or *in vitro* (Zhang et al., 2018). In addition, analysis of newly formed lymphatics following airway infection also suggests that inflammation-associated LEC junction remodeling does not require VEGFR3 signaling (Yao et al., 2012). Further studies will be required for a clear understanding of the roles of VEGFR3 signaling in LEC junction organization in developmental and disease conditions. Additionally, VEGFR3 and VEGFR2 form heterodimers in both BECs and LECs upon VEGF-A or VEGF-C stimulation (Dixelius et al., 2003; Nilsson et al., 2010), and previous studies have underlined the contribution of VEGFR2/VEGFR3 heterodimers to VEGF-C-driven tumor and corneal lymphangiogenesis and pulmonary lymphangiectasia (Yao et al., 2014; Durre et al., 2018). So far no data are available regarding the role of VEGFR2/VEGFR3 dimerization in the regulation of LEC junctions.

## Conclusion

Since functionally specialized junctions in the lymphatic endothelium were described about a decade ago, their organization and critical contribution to the uptake and transport function of lymphatic vessels have been widely recognized. However, the understanding of LEC junctions is still very limited, and many questions remain unanswered. For example, the molecular dynamics of button formation and maturation during development are poorly understood. This process is most likely to involve cytoskeletal rearrangements driven by Rho small GTPases and other effectors. In fact, the exact molecular architecture of adherens junctions and tight junctions in LEC junctions is virtually unexplored. Whether gap junctions are present at buttons and zippers and how cells communicate with each other in initial and collecting lymphatic vessels are also unknown. LECs undoubtedly share similarities with BECs in the assembly and dynamics of zipper junctions; therefore many of the intracellular partners and associated signaling components of junctional proteins identified in BECs may play similar roles in LECs. However, it is also very likely that certain LEC specific factors contribute to the unique organization and regulation of buttons, given the distinct roles of VEGF-A in opening BEC junctions and zippering LEC junctions. The involvement of these regulatory mechanisms and factors in lymphatic function and pathophysiology of inflammation, obesity and lymphedema remains to be clarified. Increasing our ability to modulate LEC junctions independently of the blood-endothelial barrier will allow to develop novel strategies against diseases characterized by dysregulation of lipid metabolism, lymph circulation and immune reactions.

## Author Contributions

FZ, GZ, and AE wrote the manuscript. SY contributed to figure preparation.

## Conflict of Interest

AE and FZ are inventors on a patent application (United States Provisional Application No. 62/873,288) submitted by the Yale University that covers compositions and methods for inhibiting dietary lipid uptake. The remaining authors declare that the research was conducted in the absence of any commercial or financial relationships that could be construed as a potential conflict of interest.

## Abbreviations

ANGPT1: angiopoietin-1
ANGPT2: angiopoietin-2
Calcrl: calcitonin receptor-like receptor
CCL21: chemokine (C-C motif) ligand 21
CCR7: C-C chemokine receptor type 7
DLL4: delta-like 4
ESAM: endothelial-specific adhesion molecule
FAK: focal adhesion kinase
FLT1: Fms-related tyrosine kinase 1
FOXC2: forkhead Box C2
ICAM-1: intercellular adhesion molecule 1
JAM-A: junctional adhesion molecule-A
LYVE-1: lymphatic vessel endothelial receptor 1
MTP: microsomal triglyceride transfer protein
NRP1: neuropilin1
NRP2: neuropilin2
PECAM-1: platelet and endothelial cell adhesion molecule 1
PROX1: prospero homeobox 1
RAMP2: receptor activity-modifying protein 2
RASIP1: Ras-interacting protein 1
ROCK: Rho-associated kinase
TAZ: tafazzin
TIE1: TEK receptor tyrosine kinase1
TIE2: TEK receptor tyrosine kinase2
TNFα: tumor necrosis factor α
VE-cadherin: vascular endothelial cadherin
VCAM-2: vascular cell adhesion molecule 2
VEGF: vascular endothelial growth factor
VEGFR: vascular endothelial growth factor receptor
VE-PTP: vascular endothelial protein tyrosine phosphatase
YAP: yes-associated protein 1
ZO-1: zonula occludens-1.

## References

[B1] Akwii R. G. Sajib M. S. Zahra F. T. Mikelis C. M. (2019). Role of angiopoietin-2 in vascular physiology and pathophysiology. *Cells* 8:E471. 10.3390/cells8050471 31108880 PMC6562915

[B2] Alitalo K. (2011). The lymphatic vasculature in disease. *Nat. Med.* 17 1371–1380. 22064427 10.1038/nm.2545

[B3] Amatschek S. Kriehuber E. Bauer W. Reininger B. Meraner P. Wolpl A. (2007). Blood and lymphatic endothelial cell-specific differentiation programs are stringently controlled by the tissue environment. *Blood* 109 4777–4785. 10.1089/lrb.2007.5106 17289814

[B4] Aspalter I. M. Gordon E. Dubrac A. Ragab A. Narloch J. Vizan P. (2015). Alk1 and Alk5 inhibition by Nrp1 controls vascular sprouting downstream of Notch. *Nat. Commun.* 6:7264. 10.1038/ncomms8264 26081042 PMC4557308

[B5] Avraham-Davidi I. Ely Y. Pham V. N. Castranova D. Grunspan M. Malkinson G. (2012). ApoB-containing lipoproteins regulate angiogenesis by modulating expression of VEGF receptor 1. *Nat. Med.* 18 967–973. 10.1038/nm.2759 22581286 PMC3959651

[B6] Baluk P. Fuxe J. Hashizume H. Romano T. Lashnits E. Butz S. (2007). Functionally specialized junctions between endothelial cells of lymphatic vessels. *J. Exp. Med.* 204 2349–2362. 10.1084/jem.20062596 17846148 PMC2118470

[B7] Banerji S. Ni J. Wang S. X. Clasper S. Su J. Tammi R. (1999). LYVE-1, a new homologue of the CD44 glycoprotein, is a lymph-specific receptor for hyaluronan. *J. Cell Biol.* 144 789–801. 10.1083/jcb.144.4.789 10037799 PMC2132933

[B8] Benedito R. Rocha S. F. Woeste M. Zamykal M. Radtke F. Casanovas O. (2012). Notch-dependent VEGFR3 upregulation allows angiogenesis without VEGF-VEGFR2 signalling. *Nature* 484 110–114. 10.1038/nature10908 22426001

[B9] Benoit J. N. Zawieja D. C. Goodman A. H. Granger H. J. (1989). Characterization of intact mesenteric lymphatic pump and its responsiveness to acute edemagenic stress. *Am. J. Physiol.* 257(Pt 2) H2059–H2069. 10.1152/ajpheart.1989.257.6.H2059 2603989

[B10] Bernier-Latmani J. Cisarovsky C. Demir C. S. Bruand M. Jaquet M. Davanture S. (2015). DLL4 promotes continuous adult intestinal lacteal regeneration and dietary fat transport. *J. Clin. Invest.* 125 4572–4586. 10.1172/JCI82045 26529256 PMC4665794

[B11] Bernier-Latmani J. Petrova T. V. (2017). Intestinal lymphatic vasculature: structure, mechanisms and functions. *Nat. Rev. Gastroenterol. Hepatol.* 14 510–526. 10.1038/nrgastro.2017.79 28655884

[B12] Blecharz K. G. Drenckhahn D. Forster C. Y. (2008). Glucocorticoids increase VE-cadherin expression and cause cytoskeletal rearrangements in murine brain endothelial cEND cells. *J. Cereb. Blood Flow Metab.* 28 1139–1149. 10.1038/jcbfm.2008.2 18231113

[B13] Bouvree K. Brunet I. Del Toro R. Gordon E. Prahst C. Cristofaro B. (2012). Semaphorin3A, Neuropilin-1, and PlexinA1 are required for lymphatic valve formation. *Circ. Res.* 111 437–445. 10.1161/CIRCRESAHA.112.269316 22723296 PMC3861899

[B14] Breslin J. W. (2014). Mechanical forces and lymphatic transport. *Microvasc. Res.* 96 46–54. 10.1016/j.mvr.2014.07.013 25107458 PMC4267889

[B15] Breslin J. W. Yuan S. Y. Wu M. H. (2007). VEGF-C alters barrier function of cultured lymphatic endothelial cells through a VEGFR-3-dependent mechanism. *Lymphat. Res. Biol.* 5 105–113. 10.1089/lrb.2007.1004 17935478 PMC3001341

[B16] Brice G. Ostergaard P. Jeffery S. Gordon K. Mortimer P. S. Mansour S. (2013). A novel mutation in GJA1 causing oculodentodigital syndrome and primary lymphoedema in a three generation family. *Clin. Genet.* 84 378–381. 10.1111/cge.12158 23550541

[B17] Bryan B. A. Dennstedt E. Mitchell D. C. Walshe T. E. Noma K. Loureiro R. (2010). RhoA/ROCK signaling is essential for multiple aspects of VEGF-mediated angiogenesis. *FASEB J.* 24 3186–3195. 10.1096/fj.09-145102 20400538 PMC2923346

[B18] Carmeliet P. Lampugnani M. G. Moons L. Breviario F. Compernolle V. Bono F. (1999). Targeted deficiency or cytosolic truncation of the VE-cadherin gene in mice impairs VEGF-mediated endothelial survival and angiogenesis. *Cell* 98 147–157. 10.1016/s0092-8674(00)81010-7 10428027

[B19] Casley-Smith J. R. (1962). The identification of chylomicra and lipoproteins in tissue sections and their passage into jejunal lacteals. *J. Cel. Biol.* 15 259–277. 10.1083/jcb.15.2.259 14019085 PMC2106149

[B20] Casley Smith J. R. (1965). Endothelial permeability. II. The passage of particles through the lymphatic endothelium of normal and injured ears. *Br. J. Exp. Pathol.* 46 35–49. 14295558 PMC2093686

[B21] Castorena-Gonzalez J. A. Zawieja S. D. Li M. Srinivasan R. S. Simon A. M. de Wit C. (2018). Mechanisms of connexin-related lymphedema. *Circ. Res.* 123 964–985. 10.1161/CIRCRESAHA.117.312576 30355030 PMC6771293

[B22] Cera M. R. Del Prete A. Vecchi A. Corada M. Martin-Padura I. Motoike T. (2004). Increased DC trafficking to lymph nodes and contact hypersensitivity in junctional adhesion molecule-A–deficient mice. *J. Clin. Invest.* 114 729–738. 10.1172/JCI21231 15343392 PMC514585

[B23] Cera M. R. Fabbri M. Molendini C. Corada M. Orsenigo F. Rehberg M. (2009). JAM-A promotes neutrophil chemotaxis by controlling integrin internalization and recycling. *J. Cell Sci.* 122(Pt 2) 268–277. 10.1242/jcs.037127 19118219

[B24] Cha B. Geng X. Mahamud M. R. Fu J. Mukherjee A. Kim Y. (2016). Mechanotransduction activates canonical Wnt/beta-catenin signaling to promote lymphatic vascular patterning and the development of lymphatic and lymphovenous valves. *Genes Dev.* 30 1454–1469. 10.1101/gad.282400.116 27313318 PMC4926867

[B25] Chistiakov D. A. Orekhov A. N. Bobryshev Y. V. (2016). Endothelial PECAM-1 and its function in vascular physiology and atherogenic pathology. *Exp. Mol. Pathol.* 100 409–415. 10.1016/j.yexmp.2016.03.012 27079772

[B26] Cho C. H. Koh Y. J. Han J. Sung H. K. Jong Lee H. Morisada T. (2007). Angiogenic role of LYVE-1-positive macrophages in adipose tissue. *Circ. Res.* 100 e47–e57. 10.1161/01.RES.0000259564.92792.93 17272806

[B27] Cifarelli V. Eichmann A. (2019). The intestinal lymphatic system: functions and metabolic implications. *Cell Mol. Gastroenterol. Hepatol.* 7 503–513. 10.1016/j.jcmgh.2018.12.002 30557701 PMC6396433

[B28] Conway D. E. Breckenridge M. T. Hinde E. Gratton E. Chen C. S. Schwartz M. A. (2013). Fluid shear stress on endothelial cells modulates mechanical tension across VE-cadherin and PECAM-1. *Curr. Biol.* 23 1024–1030. 10.1016/j.cub.2013.04.049 23684974 PMC3676707

[B29] Conway D. E. Coon B. G. Budatha M. Arsenovic P. T. Orsenigo F. Wessel F. (2017). VE-cadherin phosphorylation regulates endothelial fluid shear stress responses through the polarity protein LGN. *Curr Biol* 27 2219–2225.e5. 10.1016/j.cub.2017.08.064 28712573 PMC5667920

[B30] Coon B. G. Baeyens N. Han J. Budatha M. Ross T. D. Fang J. S. (2015). Intramembrane binding of VE-cadherin to VEGFR2 and VEGFR3 assembles the endothelial mechanosensory complex. *J. Cell Biol.* 208 975–986. 10.1083/jcb.201408103 25800053 PMC4384728

[B31] Cromer W. Wang W. Zawieja S. D. von der Weid P. Y. Newell-Rogers M. K. Zawieja D. C. (2015). Colonic insult impairs lymph flow, increases cellular content of the lymph, alters local lymphatic microenvironment, and leads to sustained inflammation in the rat ileum. *Inflamm. Bowel. Dis.* 21 1553–1563. 10.1097/MIB.0000000000000402 25939039 PMC4466086

[B32] Dartsch N. Schulte D. Hagerling R. Kiefer F. Vestweber D. (2014). Fusing VE-cadherin to alpha-catenin impairs fetal liver hematopoiesis and lymph but not blood vessel formation. *Mol. Cell. Biol.* 34 1634–1648. 10.1128/MCB.01526-13 24567373 PMC3993599

[B33] Davis R. B. Kechele D. O. Blakeney E. S. Pawlak J. B. Caron K. M. (2017). Lymphatic deletion of calcitonin receptor-like receptor exacerbates intestinal inflammation. *JCI Insight* 2:e92465. 10.1172/jci.insight.92465 28352669 PMC5358488

[B34] Dejana E. Orsenigo F. Molendini C. Baluk P. McDonald D. M. (2009a). Organization and signaling of endothelial cell-to-cell junctions in various regions of the blood and lymphatic vascular trees. *Cell Tissue Res.* 335 17–25. 10.1007/s00441-008-0694-5 18855014 PMC4422058

[B35] Dejana E. Tournier-Lasserve E. Weinstein B. M. (2009b). The control of vascular integrity by endothelial cell junctions: molecular basis and pathological implications. *Dev. Cell* 16 209–221. 10.1016/j.devcel.2009.01.004 19217423

[B36] Dellinger M. Hunter R. Bernas M. Gale N. Yancopoulos G. Erickson R. (2008). Defective remodeling and maturation of the lymphatic vasculature in Angiopoietin-2 deficient mice. *Dev. Biol.* 319 309–320. 10.1016/j.ydbio.2008.04.024 18514180 PMC2536689

[B37] Dellinger M. T. Brekken R. A. (2011). Phosphorylation of Akt and ERK1/2 is required for VEGF-A/VEGFR2-induced proliferation and migration of lymphatic endothelium. *PLoS One* 6:e28947. 10.1371/journal.pone.0028947 22174934 PMC3236226

[B38] Dellinger M. T. Meadows S. M. Wynne K. Cleaver O. Brekken R. A. (2013). Vascular endothelial growth factor receptor-2 promotes the development of the lymphatic vasculature. *PLoS One* 8:e74686. 10.1371/journal.pone.0074686 24023956 PMC3759473

[B39] Di Lorenzo A. Lin M. I. Murata T. Landskroner-Eiger S. Schleicher M. Kothiya M. (2013). eNOS-derived nitric oxide regulates endothelial barrier function through VE-cadherin and Rho GTPases. *J. Cell Sci.* 126(Pt 24) 5541–5552. 10.1242/jcs.115972 24046447 PMC3860306

[B40] Dicke N. Pielensticker N. Degen J. Hecker J. Tress O. Bald T. (2011). Peripheral lymphangiogenesis in mice depends on ectodermal connexin-26 (Gjb2). *J. Cell Sci.* 124(Pt 16) 2806–2815. 10.1242/jcs.084186 21807945

[B41] Dixelius J. Makinen T. Wirzenius M. Karkkainen M. J. Wernstedt C. Alitalo K. (2003). Ligand-induced vascular endothelial growth factor receptor-3 (VEGFR-3) heterodimerization with VEGFR-2 in primary lymphatic endothelial cells regulates tyrosine phosphorylation sites. *J. Biol. Chem.* 278 40973–40979. 10.1074/jbc.M304499200 12881528

[B42] Dixon J. B. (2010a). Lymphatic lipid transport: sewer or subway? *Trends Endocrinol. Metab.* 21 480–487. 10.1016/j.tem.2010.04.003 20541951 PMC2914116

[B43] Dixon J. B. (2010b). Mechanisms of chylomicron uptake into lacteals. *Ann. N. Y. Acad. Sci.* 1207(Suppl. 1) E52–E57. 10.1111/j.1749-6632.2010.05716.x 20961306 PMC3132563

[B44] Dorland Y. L. Huveneers S. (2017). Cell-cell junctional mechanotransduction in endothelial remodeling. *Cell Mol. Life Sci.* 74 279–292. 10.1007/s00018-016-2325-8 27506620 PMC5219012

[B45] Durre T. Morfoisse F. Erpicum C. Ebroin M. Blacher S. Garcia-Caballero M. (2018). uPARAP/Endo180 receptor is a gatekeeper of VEGFR-2/VEGFR-3 heterodimerisation during pathological lymphangiogenesis. *Nat. Commun.* 9:5178. 10.1038/s41467-018-07514-1 30518756 PMC6281649

[B46] Eichmann A. Simons M. (2012). VEGF signaling inside vascular endothelial cells and beyond. *Curr. Opin. Cell. Biol.* 24 188–193. 10.1016/j.ceb.2012.02.002 22366328 PMC4030755

[B47] Erjefalt J. S. Erjefalt I. Sundler F. Persson C. G. (1995). Epithelial pathways for luminal entry of bulk plasma. *Clin. Exp. Allergy* 25 187–195. 10.1111/j.1365-2222.1995.tb01025.x 7750011

[B48] Escobedo N. Oliver G. (2016). Lymphangiogenesis: origin, specification, and cell fate determination. *Annu. Rev. Cell Dev. Biol.* 32 677–691. 10.1146/annurev-cellbio-111315-124944 27298093

[B49] Escobedo N. Oliver G. (2017). The lymphatic vasculature: its role in adipose metabolism and obesity. *Cell Metab.* 26 598–609. 10.1016/j.cmet.2017.07.020 28844882 PMC5629116

[B50] Escobedo N. Proulx S. T. Karaman S. Dillard M. E. Johnson N. Detmar M. (2016). Restoration of lymphatic function rescues obesity in Prox1-haploinsufficient mice. *JCI Insight* 1:e85096. 10.1172/jci.insight.85096 26973883 PMC4786184

[B51] Fantin A. Lampropoulou A. Gestri G. Raimondi C. Senatore V. Zachary I. (2015). NRP1 regulates CDC42 activation to promote filopodia formation in endothelial tip cells. *Cell Rep.* 11 1577–1590. 10.1016/j.celrep.2015.05.018 26051942 PMC4528263

[B52] Fantin A. Vieira J. M. Plein A. Denti L. Fruttiger M. Pollard J. W. (2013). NRP1 acts cell autonomously in endothelium to promote tip cell function during sprouting angiogenesis. *Blood* 121 2352–2362. 10.1182/blood-2012-05-424713 23315162 PMC3606070

[B53] Ferrell R. E. Baty C. J. Kimak M. A. Karlsson J. M. Lawrence E. C. Franke-Snyder M. (2010). GJC2 missense mutations cause human lymphedema. *Am. J. Hum. Genet.* 86 943–948. 10.1016/j.ajhg.2010.04.010 20537300 PMC3032064

[B54] Figueroa X. F. Duling B. R. (2009). Gap junctions in the control of vascular function. *Antioxid. Redox Signal.* 11 251–266. 10.1089/ars.2008.2117 18831678 PMC2933153

[B55] Finegold D. N. Baty C. J. Knickelbein K. Z. Perschke S. Noon S. E. Campbell D. (2012). Connexin 47 mutations increase risk for secondary lymphedema following breast cancer treatment. *Clin. Cancer Res.* 18 2382–2390. 10.1158/1078-0432.CCR-11-2303 22351697 PMC3625665

[B56] Fischer H. Widdicombe J. H. (2006). Mechanisms of acid and base secretion by the airway epithelium. *J. Membr. Biol.* 211 139–150. 10.1007/s00232-006-0861-0 17091214 PMC2929530

[B57] Forster R. Schubel A. Breitfeld D. Kremmer E. Renner-Muller I. Wolf E. (1999). CCR7 coordinates the primary immune response by establishing functional microenvironments in secondary lymphoid organs. *Cell* 99 23–33. 10520991 10.1016/s0092-8674(00)80059-8

[B58] Friedl P. Mayor R. (2017). Tuning collective cell migration by cell-cell junction regulation. *Cold Spring Harb. Perspect. Biol.* 9:a029199. 10.1101/cshperspect.a029199 28096261 PMC5378050

[B59] Fritz-Six K. L. Dunworth W. P. Li M. Caron K. M. (2008). Adrenomedullin signaling is necessary for murine lymphatic vascular development. *J. Clin. Invest.* 118 40–50. 10.1089/lrb.2008.6102 18097475 PMC2147672

[B60] Fukuda D. Aikawa E. Swirski F. K. Novobrantseva T. I. Kotelianski V. Gorgun C. Z. (2012). Notch ligand delta-like 4 blockade attenuates atherosclerosis and metabolic disorders. *Proc. Natl. Acad. Sci. U.S.A.* 109 E1868–E1877. 10.1073/pnas.1116889109 22699504 PMC3390871

[B61] Gale N. W. Prevo R. Espinosa J. Ferguson D. J. Dominguez M. G. Yancopoulos G. D. (2007). Normal lymphatic development and function in mice deficient for the lymphatic hyaluronan receptor LYVE-1. *Mol. Cell Biol.* 27 595–604. 10.1128/MCB.01503-06 17101772 PMC1800809

[B62] Gale N. W. Thurston G. Hackett S. F. Renard R. Wang Q. McClain J. (2002). Angiopoietin-2 is required for postnatal angiogenesis and lymphatic patterning, and only the latter role is rescued by Angiopoietin-1. *Dev. Cell* 3 411–423. 10.1016/s1534-5807(02)00217-4 12361603

[B63] Gelfand M. V. Hagan N. Tata A. Oh W. J. Lacoste B. Kang K. T. (2014). Neuropilin-1 functions as a VEGFR2 co-receptor to guide developmental angiogenesis independent of ligand binding. *eLife* 3:e03720. 10.7554/eLife.03720 25244320 PMC4197402

[B64] Geng X. Cha B. Mahamud M. R. Lim K. C. Silasi-Mansat R. Uddin M. K. M. (2016). Multiple mouse models of primary lymphedema exhibit distinct defects in lymphovenous valve development. *Dev. Biol.* 409 218–233. 10.1016/j.ydbio.2015.10.022 26542011 PMC4688075

[B65] Gerli R. Solito R. Weber E. Agliano M. (2000). Specific adhesion molecules bind anchoring filaments and endothelial cells in human skin initial lymphatics. *Lymphology* 33 148–157. 11191655

[B66] Goodenough D. A. Paul D. L. (2009). Gap junctions. *Cold Spring Harb. Perspect. Biol.* 1:a002576. 10.1101/cshperspect.a002576 20066080 PMC2742079

[B67] Gordon E. J. Gale N. W. Harvey N. L. (2008). Expression of the hyaluronan receptor LYVE-1 is not restricted to the lymphatic vasculature; LYVE-1 is also expressed on embryonic blood vessels. *Dev. Dyn.* 237 1901–1909. 10.1002/dvdy.21605 18570254

[B68] Gory-Faure S. Prandini M. H. Pointu H. Roullot V. Pignot-Paintrand I. Vernet M. (1999). Role of vascular endothelial-cadherin in vascular morphogenesis. *Development* 126 2093–2102. 10207135 10.1242/dev.126.10.2093

[B69] Greene C. Hanley N. Campbell M. (2019). Claudin-5: gatekeeper of neurological function. *Fluids Barriers CNS* 16:3. 10.1186/s12987-019-0123-z 30691500 PMC6350359

[B70] Hagerling R. Hoppe E. Dierkes C. Stehling M. Makinen T. Butz S. (2018). Distinct roles of VE-cadherin for development and maintenance of specific lymph vessel beds. *EMBO J.* 37:e98271. 10.15252/embj.201798271 30297530 PMC6236332

[B71] Hagerling R. Pollmann C. Andreas M. Schmidt C. Nurmi H. Adams R. H. (2013). A novel multistep mechanism for initial lymphangiogenesis in mouse embryos based on ultramicroscopy. *EMBO J.* 32 629–644. 10.1038/emboj.2012.340 23299940 PMC3590982

[B72] Harvey N. L. Srinivasan R. S. Dillard M. E. Johnson N. C. Witte M. H. Boyd K. (2005). Lymphatic vascular defects promoted by Prox1 haploinsufficiency cause adult-onset obesity. *Nat. Genet.* 37 1072–1081. 10.1038/ng1642 16170315

[B73] Heinolainen K. Karaman S. D’Amico G. Tammela T. Sormunen R. Eklund L. (2017). VEGFR3 modulates vascular permeability by controlling VEGF/VEGFR2 signaling. *Circ. Res.* 120 1414–1425. 10.1161/CIRCRESAHA.116.310477 28298294 PMC6959003

[B74] Hellstrom M. Phng L. K. Hofmann J. J. Wallgard E. Coultas L. Lindblom P. (2007). Dll4 signalling through Notch1 regulates formation of tip cells during angiogenesis. *Nature* 445 776–780. 10.1038/nature05571 17259973

[B75] Hirakawa S. Hong Y. K. Harvey N. Schacht V. Matsuda K. Libermann T. (2003). Identification of vascular lineage-specific genes by transcriptional profiling of isolated blood vascular and lymphatic endothelial cells. *Am. J. Pathol.* 162 575–586. 10.1089/1539685041690427 12547715 PMC1851142

[B76] Hiratsuka S. Minowa O. Kuno J. Noda T. Shibuya M. (1998). Flt-1 lacking the tyrosine kinase domain is sufficient for normal development and angiogenesis in mice. *Proc. Natl. Acad. Sci. U.S.A.* 95 9349–9354. 10.1073/pnas.95.16.9349 9689083 PMC21341

[B77] Hiratsuka S. Nakao K. Nakamura K. Katsuki M. Maru Y. Shibuya M. (2005). Membrane fixation of vascular endothelial growth factor receptor 1 ligand-binding domain is important for vasculogenesis and angiogenesis in mice. *Mol. Cell. Biol.* 25 346–354. 10.1128/MCB.25.1.346-354.2005 15601855 PMC538773

[B78] Huggenberger R. Ullmann S. Proulx S. T. Pytowski B. Alitalo K. Detmar M. (2010). Stimulation of lymphangiogenesis via VEGFR-3 inhibits chronic skin inflammation. *J. Exp. Med.* 207 2255–2269. 10.1084/jem.20100559 20837699 PMC2947063

[B79] Ilina O. Friedl P. (2009). Mechanisms of collective cell migration at a glance. *J. Cell Sci.* 122(Pt 18) 3203–3208. 10.1242/jcs.036525 19726629

[B80] Jackson D. G. (2019a). Hyaluronan in the lymphatics: the key role of the hyaluronan receptor LYVE-1 in leucocyte trafficking. *Matrix Biol.* 7 219–235. 10.1016/j.matbio.2018.02.001 29425695

[B81] Jackson D. G. (2019b). Leucocyte trafficking via the lymphatic vasculature- mechanisms and consequences. *Front. Immunol.* 10:471. 10.3389/fimmu.2019.00471 30923528 PMC6426755

[B82] Jin D. Harada K. Ohnishi S. Yamahara K. Kangawa K. Nagaya N. (2008). Adrenomedullin induces lymphangiogenesis and ameliorates secondary lymphoedema. *Cardiovasc. Res.* 80 339–345. 10.1093/cvr/cvn228 18708640

[B83] Johnson L. A. Banerji S. Lawrance W. Gileadi U. Prota G. Holder K. A. (2017). Dendritic cells enter lymph vessels by hyaluronan-mediated docking to the endothelial receptor LYVE-1. *Nat. Immunol.* 18 762–770. 10.1038/ni.3750 28504698

[B84] Johnson L. A. Clasper S. Holt A. P. Lalor P. F. Baban D. Jackson D. G. (2006). An inflammation-induced mechanism for leukocyte transmigration across lymphatic vessel endothelium. *J. Exp. Med.* 203 2763–2777. 10.1084/jem.20051759 17116732 PMC2118156

[B85] Johnson L. A. Prevo R. Clasper S. Jackson D. G. (2007). Inflammation-induced uptake and degradation of the lymphatic endothelial hyaluronan receptor LYVE-1. *J. Biol. Chem.* 282 33671–33680. 10.1074/jbc.M702889200 17884820

[B86] Johnson N. C. Dillard M. E. Baluk P. McDonald D. M. Harvey N. L. Frase S. L. (2008). Lymphatic endothelial cell identity is reversible and its maintenance requires Prox1 activity. *Genes Dev.* 22 3282–3291. 10.1101/gad.1727208 19056883 PMC2600759

[B87] Jurisic G. Maby-El Hajjami H. Karaman S. Ochsenbein A. M. Alitalo A. Siddiqui S. S. (2012). An unexpected role of semaphorin3a-neuropilin-1 signaling in lymphatic vessel maturation and valve formation. *Circ. Res.* 111 426–436. 10.1161/CIRCRESAHA.112.269399 22723300 PMC3572231

[B88] Kakei Y. Akashi M. Shigeta T. Hasegawa T. Komori T. (2014). Alteration of cell-cell junctions in cultured human lymphatic endothelial cells with inflammatory cytokine stimulation. *Lymphat. Res. Biol.* 12 136–143. 10.1089/lrb.2013.0035 25166264 PMC4170982

[B89] Kanady J. D. Dellinger M. T. Munger S. J. Witte M. H. Simon A. M. (2011). Connexin37 and Connexin43 deficiencies in mice disrupt lymphatic valve development and result in lymphatic disorders including lymphedema and chylothorax. *Dev. Biol.* 354 253–266. 10.1016/j.ydbio.2011.04.004 21515254 PMC3134316

[B90] Karkkainen M. J. Haiko P. Sainio K. Partanen J. Taipale J. Petrova T. V. (2004). Vascular endothelial growth factor C is required for sprouting of the first lymphatic vessels from embryonic veins. *Nat. Immunol.* 5 74–80. 10.1038/ni1013 14634646

[B91] Kim H. Kataru R. P. Koh G. Y. (2014). Inflammation-associated lymphangiogenesis: a double-edged sword? *J. Clin. Invest.* 124 936–942. 10.1172/JCI71607 24590279 PMC3938274

[B92] Kim M. Allen B. Korhonen E. A. Nitschke M. Yang H. W. Baluk P. (2016). Opposing actions of angiopoietin-2 on Tie2 signaling and FOXO1 activation. *J. Clin. Invest.* 126 3511–3525. 10.1172/JCI84871 27548529 PMC5004955

[B93] Klein K. R. Caron K. M. (2015). Adrenomedullin in lymphangiogenesis: from development to disease. *Cell Mol. Life Sci.* 72 3115–3126. 10.1007/s00018-015-1921-3 25953627 PMC11113374

[B94] Klotz L. Norman S. Vieira J. M. Masters M. Rohling M. Dube K. N. (2015). Cardiac lymphatics are heterogeneous in origin and respond to injury. *Nature* 522 62–67. 10.1038/nature14483 25992544 PMC4458138

[B95] Koch S. Claesson-Welsh L. (2012). Signal transduction by vascular endothelial growth factor receptors. *Cold Spring Harb. Perspect. Med.* 2:a006502. 10.1101/cshperspect.a006502 22762016 PMC3385940

[B96] Korhonen E. A. Lampinen A. Giri H. Anisimov A. Kim M. Allen B. (2016). Tie1 controls angiopoietin function in vascular remodeling and inflammation. *J. Clin. Invest.* 126 3495–3510. 10.1172/JCI84923 27548530 PMC5004934

[B97] Kulkarni R. M. Herman A. Ikegami M. Greenberg J. M. Akeson A. L. (2011). Lymphatic ontogeny and effect of hypoplasia in developing lung. *Mech. Dev.* 128 29–40. 10.1016/j.mod.2010.09.003 20932899

[B98] Lawrance W. Banerji S. Day A. J. Bhattacharjee S. Jackson D. G. (2016). Binding of hyaluronan to the native lymphatic vessel endothelial receptor LYVE-1 is critically dependent on receptor clustering and hyaluronan organization. *J. Biol. Chem.* 291 8014–8030. 10.1074/jbc.M115.708305 26823460 PMC4825007

[B99] Leak L. V. (1968). Ultrastructural studies on the lymphatic anchoring filaments. *J. Cell Biol.* 36 129–149. PMC2107348 19806699

[B100] Leak L. V. (1970). Electron microscopic observations on lymphatic capillaries and the structural components of the connective tissue-lymph interface. *Microvasc. Res.* 2 361–391. 10.1016/0026-2862(70)90031-2 5523935

[B101] Leak L. V. Burke J. F. (1966). Fine structure of the lymphatic capillary and the adjoining connective tissue area. *Am. J. Anat.* 118 785–809. 10.1002/aja.1001180308 5956107

[B102] Lee J. S. (1986). Tissue fluid pressure, lymph pressure, and fluid transport in rat intestinal villi. *Microvasc. Res.* 31 170–183. 10.1016/0026-2862(86)90032-4 3702767

[B103] Li X. Padhan N. Sjostrom E. O. Roche F. P. Testini C. Honkura N. (2016). VEGFR2 pY949 signalling regulates adherens junction integrity and metastatic spread. *Nat. Commun.* 7:11017. 10.1038/ncomms11017 27005951 PMC4814575

[B104] Lim H. Y. Lim S. Y. Tan C. K. Thiam C. H. Goh C. C. Carbajo D. (2018). Hyaluronan receptor LYVE-1-expressing macrophages maintain arterial tone through hyaluronan-mediated regulation of smooth muscle cell collagen. *Immunity* 49 326–341.e7. 10.1016/j.immuni.2018.12.009 30054204

[B105] Lim H. Y. Thiam C. H. Yeo K. P. Bisoendial R. Hii C. S. McGrath K. C. (2013). Lymphatic vessels are essential for the removal of cholesterol from peripheral tissues by SR-BI-mediated transport of HDL. *Cell Metab.* 17 671–684. 10.1016/j.cmet.2013.04.002 23663736

[B106] Liu X. Gu X. Ma W. Oxendine M. Gil H. J. Davis G. E. (2018). Rasip1 controls lymphatic vessel lumen maintenance by regulating endothelial cell junctions. *Development* 145:dev165092. 10.1242/dev.165092 30042182 PMC6141773

[B107] Lobov I. B. Renard R. A. Papadopoulos N. Gale N. W. Thurston G. Yancopoulos G. D. (2007). Delta-like ligand 4 (Dll4) is induced by VEGF as a negative regulator of angiogenic sprouting. *Proc. Natl. Acad. Sci. U.S.A.* 104 3219–3224. 10.1073/pnas.0611206104 17296940 PMC1805530

[B108] Luong M. X. Tam J. Lin Q. Hagendoorn J. Moore K. J. Padera T. P. (2009). Lack of lymphatic vessel phenotype in LYVE-1/CD44 double knockout mice. *J. Cell Physiol.* 219 430–437. 10.1002/jcp.21686 19170073 PMC2665001

[B109] Mahamud M. R. Geng X. Ho Y. C. Cha B. Kim Y. Ma J. (2019). GATA2 controls lymphatic endothelial cell junctional integrity and lymphovenous valve morphogenesis through miR-126. *Development* 146:dev184218. 10.1242/dev.184218 31582413 PMC6857586

[B110] MartIn-Fontecha A. Sebastiani S. Hopken U. E. Uguccioni M. Lipp M. Lanzavecchia A. (2003). Regulation of dendritic cell migration to the draining lymph node: impact on T lymphocyte traffic and priming. *J. Exp. Med.* 198 615–621. 10.1084/jem.20030448 12925677 PMC2194169

[B111] Martinez-Corral I. Ulvmar M. H. Stanczuk L. Tatin F. Kizhatil K. John S. W. (2015). Nonvenous origin of dermal lymphatic vasculature. *Circ. Res.* 116 1649–1654. 10.1161/CIRCRESAHA.116.306170 25737499

[B112] Matsumoto-Okazaki Y. Furuse M. Kajiya K. (2012). Claudin-5 haploinsufficiency exacerbates UVB-induced oedema formation by inducing lymphatic vessel leakage. *Exp. Dermatol.* 21 557–559. 10.1111/j.1600-0625.2012.01526.x 22716257

[B113] Matsumoto T. Bohman S. Dixelius J. Berge T. Dimberg A. Magnusson P. (2005). VEGF receptor-2 Y951 signaling and a role for the adapter molecule TSAd in tumor angiogenesis. *EMBO J.* 24 2342–2353. 10.1038/sj.emboj.7600709 15962004 PMC1173150

[B114] Meens M. J. Kutkut I. Rochemont V. Dubrot J. Kaladji F. R. Sabine A. (2017). Cx47 fine-tunes the handling of serum lipids but is dispensable for lymphatic vascular function. *PLoS One* 12:e0181476. 10.1371/journal.pone.0181476 28732089 PMC5521787

[B115] Meens M. J. Sabine A. Petrova T. V. Kwak B. R. (2014). Connexins in lymphatic vessel physiology and disease. *FEBS Lett.* 588 1271–1277. 10.1016/j.febslet.2014.01.011 24457200

[B116] Mouta Carreira C. Nasser S. M. di Tomaso E. Padera T. P. Boucher Y. Tomarev S. I. (2001). LYVE-1 is not restricted to the lymph vessels: expression in normal liver blood sinusoids and down-regulation in human liver cancer and cirrhosis. *Cancer Res.* 61 8079–8084. 11719431

[B117] Muller W. A. (2015). The regulation of transendothelial migration: new knowledge and new questions. *Cardiovasc. Res.* 107 310–320. 10.1093/cvr/cvv145 25987544 PMC4592322

[B118] Munger S. J. Davis M. J. Simon A. M. (2017). Defective lymphatic valve development and chylothorax in mice with a lymphatic-specific deletion of Connexin43. *Dev. Biol.* 421 204–218. 10.1016/j.ydbio.2016.11.017 27899284 PMC5217530

[B119] Munger S. J. Geng X. Srinivasan R. S. Witte M. H. Paul D. L. Simon A. M. (2016). Segregated Foxc2, NFATc1 and Connexin expression at normal developing venous valves, and Connexin-specific differences in the valve phenotypes of Cx37, Cx43, and Cx47 knockout mice. *Dev. Biol.* 412 173–190. 10.1016/j.ydbio.2016.02.033 26953188 PMC4826804

[B120] Nagy J. A. Vasile E. Feng D. Sundberg C. Brown L. F. Detmar M. J. (2002). Vascular permeability factor/vascular endothelial growth factor induces lymphangiogenesis as well as angiogenesis. *J. Exp. Med.* 196 1497–1506. 10.1084/jem.20021244 12461084 PMC2194262

[B121] Niessen C. M. (2007). Tight junctions/adherens junctions: basic structure and function. *J. Invest. Dermatol.* 127 2525–2532. 10.1038/sj.jid.5700865 17934504

[B122] Nilsson I. Bahram F. Li X. Gualandi L. Koch S. Jarvius M. (2010). VEGF receptor 2/-3 heterodimers detected in situ by proximity ligation on angiogenic sprouts. *EMBO J.* 29 1377–1388. 10.1038/emboj.2010.30 20224550 PMC2868571

[B123] Nitschke M. Bell A. Karaman S. Amouzgar M. Rutkowski J. M. Scherer P. E. (2017). Retrograde lymph flow leads to chylothorax in transgenic mice with lymphatic malformations. *Am. J. Pathol.* 187 1984–1997. 10.1016/j.ajpath.2017.05.009 28683257 PMC5808174

[B124] Nitta T. Hata M. Gotoh S. Seo Y. Sasaki H. Hashimoto N. (2003). Size-selective loosening of the blood-brain barrier in claudin-5-deficient mice. *J. Cell Biol.* 161 653–660. 10.1083/jcb.200302070 12743111 PMC2172943

[B125] Okamoto T. Suzuki K. (2017). The role of gap junction-mediated endothelial cell-cell interaction in the crosstalk between inflammation and blood coagulation. *Int. J. Mol. Sci.* 18:E2254. 10.3390/ijms18112254 29077057 PMC5713224

[B126] Okamoto T. Usuda H. Tanaka T. Wada K. Shimaoka M. (2019). The functional implications of endothelial gap junctions and cellular mechanics in vascular angiogenesis. *Cancers (Basel)* 11:E237. 10.3390/cancers11020237 30781714 PMC6406946

[B127] Orsenigo F. Giampietro C. Ferrari A. Corada M. Galaup A. Sigismund S. (2012). Phosphorylation of VE-cadherin is modulated by haemodynamic forces and contributes to the regulation of vascular permeability in vivo. *Nat. Commun.* 3:1208. 10.1038/ncomms2199 23169049 PMC3514492

[B128] Ostergaard P. Simpson M. A. Brice G. Mansour S. Connell F. C. Onoufriadis A. (2011). Rapid identification of mutations in GJC2 in primary lymphoedema using whole exome sequencing combined with linkage analysis with delineation of the phenotype. *J. Med. Genet.* 48 251–255. 10.1136/jmg.2010.085563 21266381

[B129] Palay S. L. Karlin L. J. (1959). An electron microscopic study of the intestinal villus. II. The pathway of fat absorption. *J. Biophys. Biochem. Cytol.* 5 373–384. 10.1083/jcb.5.3.373 13664677 PMC2224670

[B130] Pascale A. Marchesi N. Govoni S. Coppola A. Gazzaruso C. (2019). The role of gut microbiota in obesity, diabetes mellitus, and effect of metformin: new insights into old diseases. *Curr. Opin. Pharmacol.* 49 1–5. 10.1016/j.coph.2019.03.011 31015106

[B131] Petrova T. V. Koh G. Y. (2018). Organ-specific lymphatic vasculature: from development to pathophysiology. *J. Exp. Med.* 215 35–49. 10.1084/jem.20171868 29242199 PMC5748863

[B132] Pflicke H. Sixt M. (2009). Preformed portals facilitate dendritic cell entry into afferent lymphatic vessels. *J. Exp. Med.* 206 2925–2935. 10.1084/jem.20091739 19995949 PMC2806476

[B133] Podgrabinska S. Braun P. Velasco P. Kloos B. Pepper M. S. Skobe M. (2002). Molecular characterization of lymphatic endothelial cells. *Proc. Natl. Acad. Sci. U.S.A.* 99 16069–16074. 10.1089/lrb.2010.0019 12446836 PMC138566

[B134] Privratsky J. R. Newman P. J. (2014). PECAM-1: regulator of endothelial junctional integrity. *Cell Tissue Res.* 355 607–619. 10.1007/s00441-013-1779-3 24435645 PMC3975704

[B135] Randolph G. J. Miller N. E. (2014). Lymphatic transport of high-density lipoproteins and chylomicrons. *J. Clin. Invest.* 124 929–935. 10.1172/JCI71610 24590278 PMC3934183

[B136] Romero I. A. Radewicz K. Jubin E. Michel C. C. Greenwood J. Couraud P. O. (2003). Changes in cytoskeletal and tight junctional proteins correlate with decreased permeability induced by dexamethasone in cultured rat brain endothelial cells. *Neurosci. Lett.* 344 112–116. 10.1016/s0304-3940(03)00348-3 12782340

[B137] Ruan G. X. Kazlauskas A. (2012). VEGF-A engages at least three tyrosine kinases to activate PI3K/Akt. *Cell Cycle* 11 2047–2048. 10.4161/cc.20535 22647379 PMC3368856

[B138] Russo E. Nitschke M. Halin C. (2013). Dendritic cell interactions with lymphatic endothelium. *Lymphat. Res. Biol.* 11 172–182. 10.1089/lrb.2013.0008 24044757 PMC3780311

[B139] Sabine A. Agalarov Y. Maby-El Hajjami H. Jaquet M. Hagerling R. Pollmann C. (2012). Mechanotransduction, PROX1, and FOXC2 cooperate to control connexin37 and calcineurin during lymphatic-valve formation. *Dev. Cell* 22 430–445. 10.1016/j.devcel.2011.12.020 22306086

[B140] Sabine A. Bovay E. Demir C. S. Kimura W. Jaquet M. Agalarov Y. (2015). FOXC2 and fluid shear stress stabilize postnatal lymphatic vasculature. *J. Clin. Invest.* 125 3861–3877. 10.1172/JCI80454 26389677 PMC4607114

[B141] Saeki H. Moore A. M. Brown M. J. Hwang S. T. (1999). Cutting edge: secondary lymphoid-tissue chemokine (SLC) and CC chemokine receptor 7 (CCR7) participate in the emigration pathway of mature dendritic cells from the skin to regional lymph nodes. *J. Immunol.* 162 2472–2475. 10072485

[B142] Salvador E. Shityakov S. Forster C. (2014). Glucocorticoids and endothelial cell barrier function. *Cell Tissue Res.* 355 597–605. 10.1007/s00441-013-1762-z 24352805 PMC3972429

[B143] Sauter B. Foedinger D. Sterniczky B. Wolff K. Rappersberger K. (1998). Immunoelectron microscopic characterization of human dermal lymphatic microvascular endothelial cells. Differential expression of CD31, CD34, and type IV collagen with lymphatic endothelial cells vs blood capillary endothelial cells in normal human skin, lymphangioma, and hemangioma in situ. *J. Histochem. Cytochem.* 46 165–176. 10.1177/002215549804600205 9446823

[B144] Sawane M. Kajiya K. Kidoya H. Takagi M. Muramatsu F. Takakura N. (2013). Apelin inhibits diet-induced obesity by enhancing lymphatic and blood vessel integrity. *Diabetes* 62 1970–1980. 10.2337/db12-0604 23378608 PMC3661640

[B145] Schmid-Schonbein G. W. (1990). Microlymphatics and lymph flow. *Physiol. Rev.* 70 987–1028. 2217560 10.1152/physrev.1990.70.4.987

[B146] Schmid-Schonbein G. W. (2003). The second valve system in lymphatics. *Lymphat. Res. Biol.* 1 25–29; discussion 29-31. 15624318 10.1089/15396850360495664

[B147] Schmitt M. M. Megens R. T. Zernecke A. Bidzhekov K. van den Akker N. M. Rademakers T. (2014). Endothelial junctional adhesion molecule-a guides monocytes into flow-dependent predilection sites of atherosclerosis. *Circulation* 129 66–76. 10.1161/CIRCULATIONAHA.113.004149 24065611

[B148] Schulte-Merker S. Sabine A. Petrova T. V. (2011). Lymphatic vascular morphogenesis in development, physiology, and disease. *J. Cell Biol.* 193 607–618. 10.1083/jcb.201012094 21576390 PMC3166860

[B149] Secker G. A. Harvey N. L. (2015). VEGFR signaling during lymphatic vascular development: from progenitor cells to functional vessels. *Dev. Dyn.* 244 323–331. 10.1002/dvdy.24227 25399804

[B150] Shibuya M. (2006). Vascular endothelial growth factor receptor-1 (VEGFR-1/Flt-1): a dual regulator for angiogenesis. *Angiogenesis* 9 225–230. ; discussion 231, 10.1007/s10456-006-9055-8 17109193

[B151] Siekmann A. F. Lawson N. D. (2007). Notch signalling limits angiogenic cell behaviour in developing zebrafish arteries. *Nature* 445 781–784. 10.1038/nature05577 17259972

[B152] Simons M. Gordon E. Claesson-Welsh L. (2016). Mechanisms and regulation of endothelial VEGF receptor signalling. *Nat. Rev. Mol. Cell Biol.* 17 611–625. 10.1038/nrm.2016.87 27461391

[B153] Sironi M. Conti A. Bernasconi S. Fra A. M. Pasqualini F. Nebuloni M. (2006). Generation and characterization of a mouse lymphatic endothelial cell line. *Cell Tissue Res.* 325 91–100. 10.1007/s00441-006-0171-y 16534603

[B154] Song S. H. Kim K. L. Lee K. A. Suh W. (2012). Tie1 regulates the Tie2 agonistic role of angiopoietin-2 in human lymphatic endothelial cells. *Biochem. Biophys. Res. Commun.* 419 281–286. 10.1016/j.bbrc.2012.02.009 22342979

[B155] Souma T. Thomson B. R. Heinen S. Carota I. A. Yamaguchi S. Onay T. (2018). Context-dependent functions of angiopoietin 2 are determined by the endothelial phosphatase VEPTP. *Proc. Natl. Acad. Sci. U.S.A.* 115 1298–1303. 10.1073/pnas.1714446115 29358379 PMC5819405

[B156] Srinivas M. Verselis V. K. White T. W. (2018). Human diseases associated with connexin mutations. *Biochim. Biophys. Acta Biomembr.* 1860 192–201. 10.1016/j.bbamem.2017.04.024 28457858 PMC5659969

[B157] Srinivasan R. S. Dillard M. E. Lagutin O. V. Lin F. J. Tsai S. Tsai M. J. (2007). Lineage tracing demonstrates the venous origin of the mammalian lymphatic vasculature. *Genes Dev.* 21 2422–2432. 10.1089/lrb.2007.5402 17908929 PMC1993873

[B158] Stamatovic S. M. Keep R. F. Andjelkovic A. V. (2008). Brain endothelial cell-cell junctions: how to “open” the blood brain barrier. *Curr. Neuropharmacol.* 6 179–192. 10.2174/157015908785777210 19506719 PMC2687937

[B159] Stanczuk L. Martinez-Corral I. Ulvmar M. H. Zhang Y. Lavina B. Fruttiger M. (2015). cKit lineage hemogenic endothelium-derived cells contribute to mesenteric lymphatic vessels. *Cell Rep.* 10 1708–1721. 10.1016/j.celrep.2015.02.026 25772358

[B160] Suchting S. Freitas C. le Noble F. Benedito R. Breant C. Duarte A. (2007). The notch ligand delta-like 4 negatively regulates endothelial tip cell formation and vessel branching. *Proc. Natl. Acad. Sci. U.S.A.* 104 3225–3230. 10.1073/pnas.0611177104 17296941 PMC1805603

[B161] Suh S. H. Choe K. Hong S. P. Jeong S. H. Makinen T. Kim K. S. (2019). Gut microbiota regulates lacteal integrity by inducing VEGF-C in intestinal villus macrophages. *EMBO Rep.* 20:e46927. 10.15252/embr.201846927 30783017 PMC6446200

[B162] Sun Z. Li X. Massena S. Kutschera S. Padhan N. Gualandi L. (2012). VEGFR2 induces c-Src signaling and vascular permeability in vivo via the adaptor protein TSAd. *J. Exp. Med.* 209 1363–1377. 10.1084/jem.20111343 22689825 PMC3405501

[B163] Suy R. Thomis S. Fourneau I. (2016). The discovery of the lymphatic system in the seventeenth century. Part II: the discovery of Chyle vessels. *Acta Chir. Belg.* 116 329–335. 10.1080/00015458.2016.1195587 27563735

[B164] Swartz M. A. Kaipainen A. Netti P. A. Brekken C. Boucher Y. Grodzinsky A. J. (1999). Mechanics of interstitial-lymphatic fluid transport: theoretical foundation and experimental validation. *J. Biomech.* 32 1297–1307. 10.1016/s0021-9290(99)00125-6 10569708

[B165] Szymborska A. Gerhardt H. (2018). Hold me, but not too tight-endothelial cell-cell junctions in angiogenesis. *Cold Spring Harb. Perspect. Biol.* 10:a029223. 10.1101/cshperspect.a029223 28851748 PMC6071488

[B166] Tacconi C. Correale C. Gandelli A. Spinelli A. Dejana E. D’Alessio S. (2015). Vascular endothelial growth factor C disrupts the endothelial lymphatic barrier to promote colorectal cancer invasion. *Gastroenterology* 148 1438.e–1451.e. 10.1053/j.gastro.2015.03.005 25754161

[B167] Tammela T. Alitalo K. (2010). Lymphangiogenesis: molecular mechanisms and future promise. *Cell* 140 460–476. 10.1016/j.cell.2010.01.045 20178740

[B168] Tammela T. Zarkada G. Nurmi H. Jakobsson L. Heinolainen K. Tvorogov D. (2011). VEGFR-3 controls tip to stalk conversion at vessel fusion sites by reinforcing Notch signalling. *Nat. Cell Biol.* 13 1202–1213. 10.1038/ncb2331 21909098 PMC3261765

[B169] Tammela T. Zarkada G. Wallgard E. Murtomaki A. Suchting S. Wirzenius M. (2008). Blocking VEGFR-3 suppresses angiogenic sprouting and vascular network formation. *Nature* 454 656–660. 10.1038/nature07083 18594512

[B170] Teijeira A. Garasa S. Pelaez R. Azpilikueta A. Ochoa C. Marre D. (2013). Lymphatic endothelium forms integrin-engaging 3D structures during DC transit across inflamed lymphatic vessels. *J. Invest. Dermatol.* 133 2276–2285. 10.1038/jid.2013.152 23528818

[B171] Torzicky M. Viznerova P. Richter S. Strobl H. Scheinecker C. Foedinger D. (2012). Platelet endothelial cell adhesion molecule-1 (PECAM-1/CD31) and CD99 are critical in lymphatic transmigration of human dendritic cells. *J. Invest. Dermatol.* 132 1149–1157. 10.1038/jid.2011.420 22189791

[B172] Trani M. Dejana E. (2015). New insights in the control of vascular permeability: vascular endothelial-cadherin and other players. *Curr. Opin. Hematol.* 22 267–272. 10.1097/MOH.0000000000000137 25767951

[B173] Triacca V. Guc E. Kilarski W. W. Pisano M. Swartz M. A. (2017). Transcellular pathways in lymphatic endothelial cells regulate changes in solute transport by fluid stress. *Circ. Res.* 120 1440–1452. 10.1161/CIRCRESAHA.116.309828 28130294

[B174] Trincot C. E. Xu W. Zhang H. Kulikauskas M. R. Caranasos T. G. Jensen B. C. (2019). Adrenomedullin induces cardiac lymphangiogenesis after myocardial infarction and regulates cardiac edema via connexin 43. *Circ. Res.* 124 101–113. 10.1161/CIRCRESAHA.118.313835 30582443 PMC6318063

[B175] Trzewik J. Mallipattu S. K. Artmann G. M. Delano F. A. Schmid-Schonbein G. W. (2001). Evidence for a second valve system in lymphatics: endothelial microvalves. *FASEB J.* 15 1711–1717. 10.1096/fj.01-0067com 11481218

[B176] Tzima E. Irani-Tehrani M. Kiosses W. B. Dejana E. Schultz D. A. Engelhardt B. (2005). A mechanosensory complex that mediates the endothelial cell response to fluid shear stress. *Nature* 437 426–431. 10.1038/nature03952 16163360

[B177] Vaahtomeri K. Karaman S. Makinen T. Alitalo K. (2017). Lymphangiogenesis guidance by paracrine and pericellular factors. *Genes Dev.* 31 1615–1634. 10.1101/gad.303776.117 28947496 PMC5647933

[B178] van Nieuw Amerongen G. P. Koolwijk P. Versteilen A. van Hinsbergh V. W. (2003). Involvement of RhoA/Rho kinase signaling in VEGF-induced endothelial cell migration and angiogenesis in vitro. *Arterioscler. Thromb. Vasc. Biol.* 23 211–217. 10.1161/01.atv.0000054198.68894.88 12588761

[B179] Venero Galanternik M. Stratman A. N. Jung H. M. Butler M. G. Weinstein B. M. (2016). Building the drains: the lymphatic vasculature in health and disease. *Wiley Interdiscip. Rev. Dev. Biol.* 5 689–710. 10.1002/wdev.246 27576003

[B180] Vieira J. M. Norman S. Villa Del Campo C. Cahill T. J. Barnette D. N. Gunadasa-Rohling M. (2018). The cardiac lymphatic system stimulates resolution of inflammation following myocardial infarction. *J. Clin. Invest.* 128 3402–3412. 10.1172/JCI97192 29985167 PMC6063482

[B181] Vigl B. Aebischer D. Nitschke M. Iolyeva M. Rothlin T. Antsiferova O. (2011). Tissue inflammation modulates gene expression of lymphatic endothelial cells and dendritic cell migration in a stimulus-dependent manner. *Blood* 118 205–215. 10.1182/blood-2010-12-326447 21596851

[B182] Wang Y. Baeyens N. Corti F. Tanaka K. Fang J. S. Zhang J. (2016). Syndecan 4 controls lymphatic vasculature remodeling during mouse embryonic development. *Development* 143 4441–4451. 10.1242/dev.140129 27789626 PMC5201046

[B183] Wigle J. T. Oliver G. (1999). Prox1 function is required for the development of the murine lymphatic system. *Cell* 98 769–778. 10.1016/s0092-8674(00)81511-1 10499794

[B184] Williams C. K. Li J. L. Murga M. Harris A. L. Tosato G. (2006). Up-regulation of the notch ligand delta-like 4 inhibits VEGF-induced endothelial cell function. *Blood* 107 931–939. 10.1182/blood-2005-03-1000 16219802 PMC1895896

[B185] Wirzenius M. Tammela T. Uutela M. He Y. Odorisio T. Zambruno G. (2007). Distinct vascular endothelial growth factor signals for lymphatic vessel enlargement and sprouting. *J. Exp. Med.* 204 1431–1440. 10.1084/jem.20062642 17535974 PMC2118625

[B186] Wong H. L. Jin G. Cao R. Zhang S. Cao Y. Zhou Z. (2016). MT1-MMP sheds LYVE-1 on lymphatic endothelial cells and suppresses VEGF-C production to inhibit lymphangiogenesis. *Nat. Commun.* 7:10824. 10.1038/ncomms10824 26926389 PMC4773521

[B187] Woodfin A. Reichel C. A. Khandoga A. Corada M. Voisin M. B. Scheiermann C. (2007). JAM-A mediates neutrophil transmigration in a stimulus-specific manner in vivo: evidence for sequential roles for JAM-A and PECAM-1 in neutrophil transmigration. *Blood* 110 1848–1856. 10.1182/blood-2006-09-047431 17505016

[B188] Xu H. Chen M. Reid D. M. Forrester J. V. (2007). LYVE-1-positive macrophages are present in normal murine eyes. *Invest. Ophthalmol. Vis. Sci.* 48 2162–2171. 10.1167/iovs.06-0783 17460275

[B189] Xu Y. Yuan L. Mak J. Pardanaud L. Caunt M. Kasman I. (2010). Neuropilin-2 mediates VEGF-C-induced lymphatic sprouting together with VEGFR3. *J. Cell Biol.* 188 115–130. 10.1083/jcb.200903137 20065093 PMC2812843

[B190] Yang Y. Cha B. Motawe Z. Y. Srinivasan R. S. Scallan J. P. (2019). VE-cadherin is required for lymphatic valve formation and maintenance. *Cell Rep.* 28 2397–2412.e4. 10.1016/j.celrep.2019.07.072 31461654 PMC6743082

[B191] Yang Y. Garcia-Verdugo J. M. Soriano-Navarro M. Srinivasan R. S. Scallan J. P. Singh M. K. (2012). Lymphatic endothelial progenitors bud from the cardinal vein and intersomitic vessels in mammalian embryos. *Blood* 120 2340–2348. 10.1182/blood-2012-05-428607 22859612 PMC3447786

[B192] Yao L. C. Baluk P. Feng J. McDonald D. M. (2010). Steroid-resistant lymphatic remodeling in chronically inflamed mouse airways. *Am. J. Pathol.* 176 1525–1541. 10.2353/ajpath.2010.090909 20093490 PMC2832171

[B193] Yao L. C. Baluk P. Srinivasan R. S. Oliver G. McDonald D. M. (2012). Plasticity of button-like junctions in the endothelium of airway lymphatics in development and inflammation. *Am. J. Pathol.* 180 2561–2575. 10.1016/j.ajpath.2012.02.019 22538088 PMC3378913

[B194] Yao L. C. Testini C. Tvorogov D. Anisimov A. Vargas S. O. Baluk P. (2014). Pulmonary lymphangiectasia resulting from vascular endothelial growth factor-C overexpression during a critical period. *Circ. Res.* 114 806–822. 10.1161/CIRCRESAHA.114.303119 24429550 PMC3969887

[B195] Yazdani S. Jaldin-Fincati J. R. Pereira R. V. S. Klip A. (2019). Endothelial cell barriers: transport of molecules between blood and tissues. *Traffic* 20 390–403. 10.1111/tra.12645 30950163

[B196] Yuan Y. Arcucci V. Levy S. M. Achen M. G. (2019). Modulation of immunity by lymphatic dysfunction in lymphedema. *Front. Immunol.* 10:76. 10.3389/fimmu.2019.00076 30761143 PMC6361763

[B197] Zarkada G. Heinolainen K. Makinen T. Kubota Y. Alitalo K. (2015). VEGFR3 does not sustain retinal angiogenesis without VEGFR2. *Proc. Natl. Acad. Sci. U.S.A.* 112 761–766. 10.1073/pnas.1423278112 25561555 PMC4311859

[B198] Zhang F. Zarkada G. Han J. Li J. Dubrac A. Ola R. (2018). Lacteal junction zippering protects against diet-induced obesity. *Science* 361 599–603. 10.1126/science.aap9331 30093598 PMC6317738

[B199] Zheng W. Nurmi H. Appak S. Sabine A. Bovay E. Korhonen E. A. (2014). Angiopoietin 2 regulates the transformation and integrity of lymphatic endothelial cell junctions. *Genes Dev.* 28 1592–1603. 10.1101/gad.237677.114 25030698 PMC4102766

[B200] Zhou Q. Wood R. Schwarz E. M. Wang Y. J. Xing L. (2010). Near-infrared lymphatic imaging demonstrates the dynamics of lymph flow and lymphangiogenesis during the acute versus chronic phases of arthritis in mice. *Arthritis Rheum.* 62 1881–1889. 10.1002/art.27464 20309866 PMC2902657

